# Global Proteomics Analysis of Bone Marrow: Establishing Talin-1 and Centrosomal Protein of 55 kDa as Potential Molecular Signatures for Myelodysplastic Syndromes

**DOI:** 10.3389/fonc.2022.833068

**Published:** 2022-06-22

**Authors:** Arlindo A. Moura, Maria Julia B. Bezerra, Aline M. A. Martins, Daniela P. Borges, Roberta T. G. Oliveira, Raphaela M. Oliveira, Kaio M. Farias, Arabela G. Viana, Guilherme G. C. Carvalho, Carlos R. K. Paier, Marcelo V. Sousa, Wagner Fontes, Carlos A. O. Ricart, Maria Elisabete A. Moraes, Silvia M. M. Magalhães, Cristiana L. M. Furtado, Manoel O. Moraes-Filho, Claudia Pessoa, Ronald F. Pinheiro

**Affiliations:** ^1^ Graduate Program in Animal Science, Federal University of Ceará, Fortaleza, Brazil; ^2^ Drug Research and Development Center (NPDM), The School of Medicine, Federal University of Ceará, Fortaleza, Brazil; ^3^ Graduate Program in Biotechnology (Renorbio), Federal University of Ceará, Fortaleza, Brazil; ^4^ Laboratory of Protein Chemistry and Biochemistry, The University of Brasília, Brasília, Brazil; ^5^ Graduate Program in Medical Sciences, The School of Medicine, Federal University of Ceará, Fortaleza, Brazil; ^6^ Graduate Program in Pharmacology, Federal University of Ceará, Fortaleza, Brazil; ^7^ Graduate Program in Translational Medicine, The School of Medicine, Federal University of Ceará, Fortaleza, Brazil; ^8^ Experimental Biology Center, NUBEX, The University of Fortaleza (Unifor), Fortaleza, Brazil

**Keywords:** myelodysplastic syndrome, centrosomal protein of 55 kDa (CEP55), Talin-1 (TLN1), bone marrow, karyotype, proteomics

## Abstract

Myelodysplastic syndrome (MDS) is a hematological disorder characterized by abnormal stem cell differentiation and a high risk of acute myeloid leukemia transformation. Treatment options for MDS are still limited, making the identification of molecular signatures for MDS progression a vital task. Thus, we evaluated the proteome of bone marrow plasma from patients (n = 28) diagnosed with MDS with ring sideroblasts (MDS-RS) and MDS with blasts in the bone marrow (MDS-EB) using label-free mass spectrometry. This strategy allowed the identification of 1,194 proteins in the bone marrow plasma samples. Polyubiquitin-C (UBC), moesin (MSN), and Talin-1 (TLN1) showed the highest abundances in MDS-EB, and centrosomal protein of 55 kDa (CEP55) showed the highest relative abundance in the bone marrow plasma of MDS-RS patients. In a follow-up, in the second phase of the study, expressions of *UBC*, *MSN*, *TLN1*, and *CEP55* genes were evaluated in bone marrow mononuclear cells from 45 patients by using qPCR. This second cohort included only seven patients from the first study. *CEP55*, *MSN*, and *UBC* expressions were similar in mononuclear cells from MDS-RS and MDS-EB individuals. However, *TLN1* gene expression was greater in mononuclear cells from MDS-RS (p = 0.049) as compared to MDS-EB patients. Irrespective of the MDS subtype, *CEP55* expression was higher (p = 0.045) in MDS patients with abnormal karyotypes, while *MSN*, *UBC*, and *TALIN1* transcripts were similar in MDS with normal vs. abnormal karyotypes. In conclusion, proteomic and gene expression approaches brought evidence of altered TLN1 and CEP55 expressions in cellular and non-cellular bone marrow compartments of patients with low-risk (MDS-RS) and high-risk (MDS-EB) MDSs and with normal vs. abnormal karyotypes. As MDS is characterized by disrupted apoptosis and chromosomal alterations, leading to mitotic slippage, TLN1 and CEP55 represent potential markers for MDS prognosis and/or targeted therapy.

## 1 Introduction

Myelodysplastic syndromes (MDSs) are hematologic stem cell malignancies associated with cytopenias, bone marrow insufficiency, and anemia (1). The pathogenesis of MDS relates to disruption of stem cell development, increased apoptosis, mutations in splicing factors and DNA repair system, altered DNA methylation of tumor suppressor genes or proto-oncogenes, and immune derangement (2–4). According to the Revised International Prognostic Scoring System (IPSS-R), MDS is grouped into very low-, low-, intermediate-, very high-, and high-risk disorders, depending on the pathogenesis and risk of acute myeloid leukemia (AML) transformation (5).

MDS with ring sideroblasts (MDS-RS) is characterized by bone marrow dysplasia, cytopenia, low risk of AML transformation, ring sideroblasts ≥15% of bone marrow precursors, and presence of *SF3B1* mutation (6, 7). Ring sideroblasts are erythroid precursors with abnormal accumulation of iron inside mitochondria, and MDS-RS cases have stem cells with abnormal erythroid colony formation with failure of terminal erythroid differentiation (TED) (8). TED was also reported in high-risk MDS (8), characterized by excess blasts (MDS-EB) and 5%–19% myeloblasts in the bone marrow and accounts for up to 40% of all MDS cases, with a high risk of AML transformation (6, 9, 10). Low-risk MDS, such as MDS-RS, commonly presents symptoms of anemia and proper response to erythropoietin treatment (8). MDS-EB is linked to high-risk karyotypes such as monosomy seven, complex karyotype and mutations of genes that control disturbance of apoptosis (*TP53*) and DNA repair, stem cell maturation (*RUNX1*), and histone modifications (*ASXL1*) (11).

The bone marrow microenvironment contains cellular and non-cellular compartments that modulate the functional status of hematopoietic stem cells (12), and soluble factors present in the bone marrow modulate cell–cell and cell–extracellular matrix interactions and intracellular signaling (13). Alterations in their biochemistry and synthesis of bone marrow factors interfere with hematopoiesis and potentially trigger aberrant mechanisms that define the pathobiology of MDS. In fact, a recent study reports that pronounced differences exist in the proteomes of bone marrow plasma of patients with AML and healthy ones (14), with upregulated and downregulated proteins associated with chemokine and cytokine signaling pathways. Protein profiles are also altered in the blood and bone marrow plasma of patients with low- and high-risk lymphocytic leukemia (Braoudaki et al. (15)). The proteome of bone marrow cellular compartments and blood plasma are also altered in patients with AML (16), and plasma from patients diagnosed with MDS has decreased levels of CXC chemokine ligands 4 and 7 (17). Analyses of plasma samples using 2-D sodium dodecyl sulfate–polyacrylamide gel electrophoresis (SDS-PAGE) and nano-liquid chromatography–tandem mass spectrometry (nano-LC-MS/MS) identified 39 proteins with different abundances in MDS patients with refractory anemia and refractory anemia with ringed sideroblasts (18). The same research team reported 27 different proteins in the plasma of MDS patients with refractory anemia with excess blasts-2 in comparison to healthy individuals (19). Other studies used gel-based proteomics to describe protein profiles of platelets (20) and peripheral mononuclear cells (21) of patients with MDS. These results clearly indicate that hematological stem cell malignancies are preceded by and/or cause dramatic alterations in the proteome of diverse biological entities, from blood to components of the bone marrow.

Researchers have made remarkable contributions to the understanding of the pathophysiology of MDSs (4, 22–24). Despite this unprecedented effort, the current therapy with hypomethylating agents is not effective for all cases of the disease, especially for high-risk MDS (22, 25), and the identification of molecular markers of MDS progression and response to treatment may be crucial for effective therapeutic strategies in precision medicine. Thus, a series of studies were designed to decipher potential molecular signatures of MDS subtypes. The first study evaluated the proteome of bone marrow plasma from patients diagnosed with low-risk (MDS-RS) and high-risk MDS (MDS-EB). As centrosomal protein of 55 kDa (CEP55), moesin (MSN), talin-1 (TLN1), and ubiquitin C (UBC) showed the highest differential abundances in MDS-RS vs. MDS-EB cases, a second phase of the research was carried out to analyze the expression of those respective genes in mononuclear cells of a distinct cohort of MDS patients.

## 2 Materials and Methods

### 2.1 Research Design and Ethical Statement

**Figure 1:**
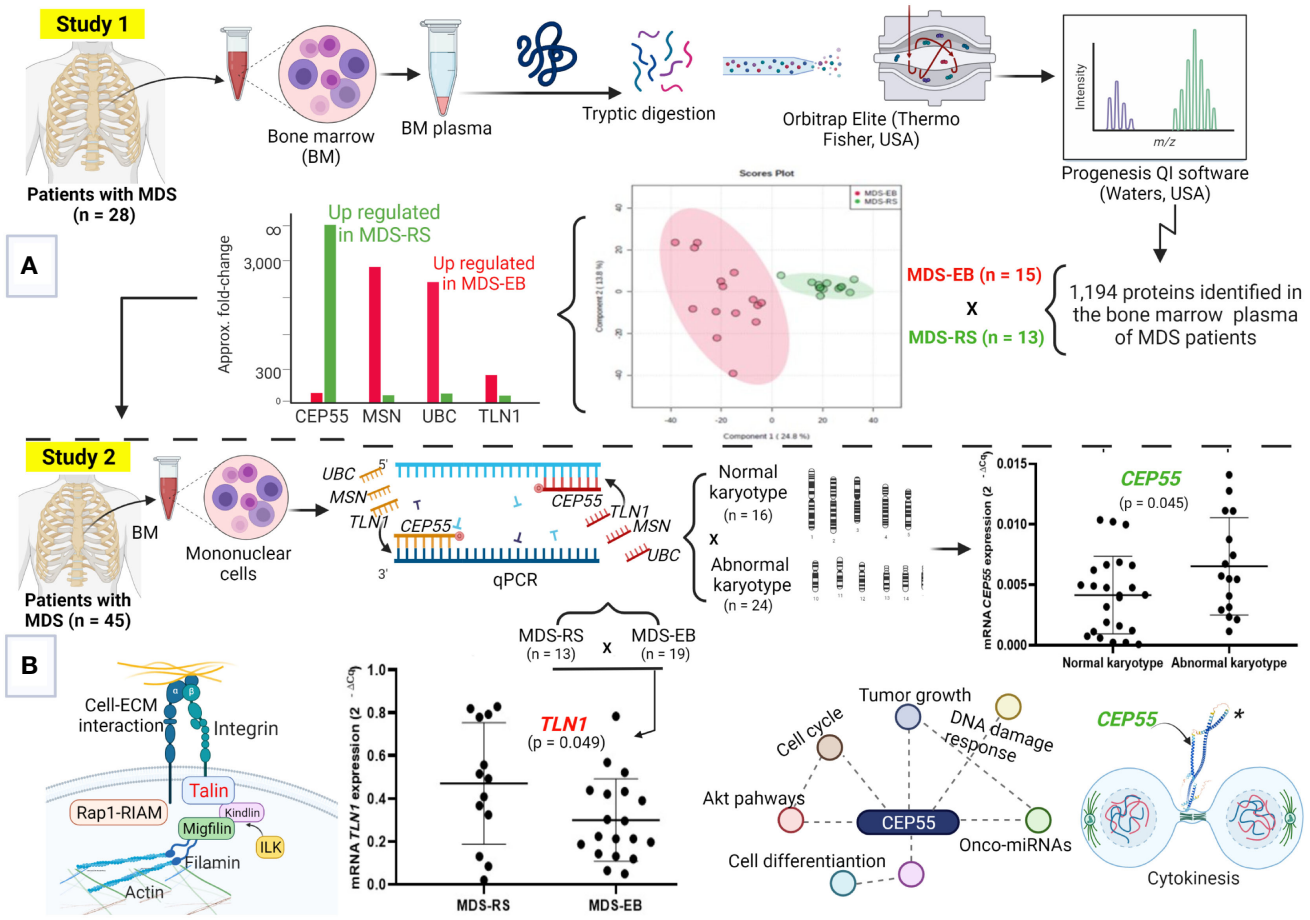
Molecular signatures of myelodysplastic syndromes (MDS). **(A)** Study 1, focused on the proteome of bone marrow (BM) plasma from patients with MDS-RS and MDS-EB. This strategy defined the highest abundances of UBC, MSN, and TLN1 in MDS-EB and CEP55, in the BM plasma of MDS-RS patients. **(B)** The steps of Study 2, where *UBC*, *MSN*, *TLN1*, and *CEP55* genes were evaluated in MDS patients. Analyses by qPCR show greater *TLN1* expression in mononuclear cells of MDS-RS vs. MDS-EB patients, and *CEP55* is associated with abnormal karyotypes. MDS-RS, MDS with ring sideroblasts; MDS-EB, MDS with excess blasts. Figure generated using BioRender (https://biorender.com), and CEP55 structure* was downloaded from “AlphaFold Protein Structure Database” (https://alphafold.ebi.ac.uk/entry/Q53EZ4).

The present research consisted of two independent studies (**Figure 1**). In Study 1, label-free data-dependent acquisition (DDA) mass spectrometry was used to decipher the proteome of bone marrow plasma from patients with MDS-RS and MDS-EB (n = 28). Tools of bioinformatics and statistical methods were employed for *in silico* analysis of protein functional attributes and protein differential quantities in the samples. Based on such analyses, CEP55, MSN, TLN1, and UBC were proteins with the highest differential abundances in the bone marrow plasma of MDS-RS and MDS-EB patients. Thus, Study 2 was further carried out to examine the expression of those genes in bone marrow mononuclear cells from an additional cohort of MDS patients (n = 45).

This study was approved by the Ethics Committee of the Federal University of Ceará, UFC (#69320817.7.0000.5054), with written consent from each participant. Patients were diagnosed with MDS at the Drug Research and Development Center, School of Medicine, UFC, and corresponded to treatment-naive patients.

### 2.2 Cytogenetics

G-banding karyotype of patients from Study 1 and Study 2 (see below) was performed as previously reported (26). Briefly, cultures were established in Roswell Park Memorial Institute (RPMI) 1640 medium (Gibco, Grand Island, NY, USA) containing 30% fetal calf serum. For the 24-h culture, colcemid was added at a 0.05 μg/ml concentration for the final 30 min of culture. After being harvested, cells were exposed to a hypotonic KCl solution (0.068 mol/L) and fixed with Carnoy’s buffer (1:3 acetic acid/methanol). Slides were stained with Giemsa solution, and 20 metaphases were analyzed whenever possible. The karyotype was prepared using CytoVision Automated Karyotyping System (Applied Imaging, Grand Rapids, MI, USA) and described according to the International System for Human Cytogenomic Nomenclature 2016 (27).

#### 2.2.1 Study 1: Quantitative Proteomic Analysis of Bone Marrow Plasma

The proteome of bone marrow plasma was evaluated in samples from 13 patients with MDS-RS and 15 patients diagnosed with MDS-EB, which included subcategories MDS-EB-1 (5%–9% blasts in the bone marrow) and MDS-EB-2 (10%–19% blasts in the bone marrow) (**Table 1**). Diagnosis of the disease followed the WHO 2016 guidelines (1), and the risk stratification was based on the IPSS-R (5).

**Table 1:** Summary of clinical and prognostic characteristics of patients with myelodysplastic syndrome (MDS) with ring sideroblasts (MDS-RS) and MDS with excess blasts (MDS-EB).

Case	Gender	Age	Hb (g/dL)	ANC (/mm³)	Platelets (/mm³)	Blasts (%)	Karyotype	WHO 2016	IPSS-R
1	M	76	9	1.70	184.00	0	45,X.-Y[18]/46.XY[7]	MDS-RS	Very Low
2	F	72	4.4	1.339	101.00	1	No metaphase	MDS-RS	–
3	M	85	6.7	2.209	171.00	1	46,XY[15]	MDS-RS	Low
4	F	66	6.5	2.293	262.00	4	No metaphase	MDS-RS	–
5	F	75	8.7	3.391	382.00	1	46,XX[15]	MDS-RS	Low
6	M	60	6.12	3.643	51.83	2	No metaphase	MDS-RS	–
7	F	73	7.5	3.844	47.210	1	No metaphase	MDS-RS	–
8	F	82	5.87	2.180	267.00	2	46,XX[5]	MDS-RS	Low
9	F	70	8.07	2.294	499.00	0	No metaphase	MDS-RS	–
10	F	73	9.76	5.106	676.00	0	46,XX[12]	MDS-RS	Low
11	M	82	7.63	3.599	338.00	0	46,XX[20]	MDS-RS	Low
12	M	85	7.5	2.726	334.10	0.5	No metaphase	MDS-RS	–
13	F	87	10.9	2.728	292.00	1	No metaphase	MDS-RS	–
14	F	60	9.2	217	116.00	5	No metaphase	MDS-EB	–
15	M	59	7.7	222	31.50	5	No metaphase	MDS-EB	–
16	F	75	11	1.548	124.00	5	No metaphase	MDS-EB	–
17	M	72	7.2	813	47.00	5	No metaphase	MDS-EB	–
18	M	84	9.1	344	33.00	18	No metaphase	MDS-EB	–
19	M	58	7.2	275	22.00	18	47,XY,+8[6]/47,XY,del(7)(q32),+8[7]/46,XY[2]	MDS-EB	Very High
20	M	63	7.5	169	51.00	9	46,XY[4]	MDS-EB	High
21	M	79	7.2	1.045	87.00	19	No metaphase	MDS-EB	–
22	F	57	6	1,028	460.00	5	46,XX[10]	MDS-EB	High
23	F	86	6.7	1,280	148.00	19	46,XX[8]	MDS-EB	High
24	M	73	7.1	460	26.00	12	No metaphase	MDS-EB	–
25	M	80	11	938	128.00	8	47,XY,+8[12]/46,XY[8]	MDS-EB	Intermediate
26	M	68	5.61	289	24.00	17	46,XY[5]	MDS-EB	Very High
27	F	44	6.76	1,887	9.98	15	46,XX[20]	MDS-EB	Very High
28	M	74	12.7	843	98.00	10	46,XY,del(17)(p11.2)[3]/47,XY,+mar[3]/47,XY,+20[4]/46,XY,del(17)(p11.2),+mar[2]/46,XY[9]	MDS-EB	High

ANC, Absolut Neutrophil Count; F, Female; Hb, Hemoglobin; IPSS-R, Revised International Prognostic Score System; M, Male.

##### 2.2.1.1 Sample Collection and Preparation, Trypsinization, and Desalting

Samples were obtained by sternal aspiration of bone marrow by a clinical hematologist at the same time as trephine biopsy at diagnosis. The bone marrow was collected in 4-ml Vacutainer™ tubes with spray-coated K_2_EDTA (Thermo Fisher, Waltham, MA, USA). Immediately after collection, tubes were inverted several times and centrifuged at 700 ×*g* (15 min, 4°C). Then, the supernatant (plasma) was transferred to another tube, treated with a protease inhibitor mix (Sigma-Aldrich, St. Louis, MO, USA), and centrifuged at 10,000 ×*g* (30* min*, 4°C). Afterward, the plasma was pipetted into a clean tube and stored at −80°C. An aliquot of this plasma was used for soluble protein quantification (28). Based on this quantification, both 1-D and 2-D gels (SDS-PAGE) were run for assessment of general protein patterns (**Supplementary Figure 1**). Gels were prepared and run as reported before (29), with modifications.

Bone marrow plasma proteins (25 µg) were dissolved in a solubilization buffer (8 M of urea, 0.02 M of TEAB, and 0.5 M of dithiothreitol), followed by incubation at 55°C (400 rpm agitation, 25 min). Then, iodoacetamide was added (0.014 M), and the mixture was maintained at 21°C in the dark (400 rpm, 40 min). Next, buffer (0.005 M of dithiothreitol, 0.001 M of CaCl_2_, and 0.02 M of TEAB) was added to reach 75-μl volume. Samples were incubated with trypsin (Promega, Madison, WI, USA) at 37°C for 18 h, and trifluoroacetic acid (1%) was added to stop the tryptic activity. Then, stage tip C18 columns were made using Empore™ SPE disks for peptide desalting (Sigma-Aldrich, Darmstadt, Germany), as reported before (29, 30). Trypsin-digested samples were added to the columns, washed, and eluted with acetonitrile (0.5%; 25% to 80%) and 0.5% acetic acid. Peptides were quantified prior to mass spectrometry analysis (Qubit™; Thermo Fisher, USA).

##### 2.2.1.2 Label-Free Mass Spectrometry

Three micrograms of tryptic peptides from each sample was individually applied to a Dionex Ultimate 3000 liquid chromatographer (Thermo Scientific, USA) for reversed-phase nano-chromatography, as previously described (31). The peptides were injected into a 2 cm × 100 μm trap column containing C18, 5-μm particles (Dr. Maisch GmbH, Ammerbuch, Germany). Then, peptides were eluted from this column to another analytical one (32 cm × 75 μm) containing C18, 3-μm particles (Dr. Maisch GmbH, Germany), and finally eluted to the spectrometer’s ionization source. The elution gradient was composed of 0.1% formic acid in water (solvent A) and 0.1% formic acid in acetonitrile (solvent B), in a gradient of 2% to 35% solvent B for 170 min.

Samples were analyzed in positive DDA mode in a label-free mass spectrometric approach using an Orbitrap Elite instrument (Thermo Fisher, USA) (31, 32). The eluted fractions generated MS1 spectra between 300 and 1,650 *m*/*z* with a resolution of 120,000 FWHM at 400 *m*/*z*. The 20 most abundant ions from MS1 with at least two charges were automatically selected to fragmentation (MS2) by higher-energy collisional dissociation (HCD) with automatic gain control (AGC) of 1 × 10 (6) and dynamic exclusion of 10 ppm for 90 s. HCD isolation window was set for 2.0 *m*/*z*, with 5 × 10 (4) AGC, normalized collision energy of 35%, and threshold for fragmentation of 3,000. The datasets for this study can be found in the Repository MassIVE https://massive.ucsd.edu/ProteoSAFe/static/massive.jsp MassIVE ID=MSV000088457.

##### 2.2.1.3 Data Analyses

MS1 spectra found in the chromatograms were aligned and quantified according to integrated intensity area from the extracted-ion chromatogram (XIC) peaks generated by the respective ion and quantified using Progenesis QI software Nonlinear Dynamics (Waters, Milford, MA, USA). Protein identification was performed using Peaks software, which deduces sequences from the fragmentation information and searches in the UniProt database. Protein identification information was inserted again in the Progenesis QI program and combined with quantitative data generated previously (31).

Statistical analysis was performed using Progenesis QI software to evaluate differences in protein abundance between MDS-RS and MDS-EB samples. A first statistical analysis was performed before protein identification to filter the MS1 features presenting ANOVA p-values <0.05. Peaks 7.0 software was used with the fragmentation spectra, and the *Homo sapiens* UniProt database was searched. Search parameters were set as follows: precursor ion mass error tolerance of 10 ppm, MS/MS mass tolerance of 0.05 Da, carbamidomethylation of cysteine residues (fixed modification), deamidation, and methionine oxidation (variable modifications). A maximum of two missed cleavage sites per peptide was allowed. The identified proteins were filtered at a rate of 1% for false discovery rate (FDR), and a minimum of 1 unique peptide per protein was required for identification. Proteins with different abundances (p < 0.05) between MDS-RS and MDS-EB samples were subjected to multivariate analysis using MetaboAnalyst 4.0 (http://www.metaboanalyst.ca) (31, 33, 34). For this type of analysis, protein data were log transformed, normalized based on sample median, and mean centered. Principal component analysis and partial-least squares discriminant analysis (PLS-DA) with variable importance in projection (VIP) approaches were applied to verify data trends and outliers, protein discrimination, and importance in the MDS-RS vs. MDS-EB scenario, respectively.

##### 2.2.1.4 Analysis of Functional Clusters

Functional clusters associated with bone marrow plasma proteins of patients diagnosed with MDS-RS and MDS-EB were analyzed through the DAVID platform (DAVID—Functional Annotation Bioinformatics Analysis—https://david.ncifcrf.gov) (35–37). For this analysis, UniProt accession numbers were uploaded to the DAVID platform, and clusters were defined according to enrichment scores and p-values [−log (p-value)].

#### 2.2.2 Study 2: Analysis of Gene Expressions in Bone Marrow Mononuclear Cells

Based on the results of Study 1, UBC, MSN, and TLN1 proteins showed the highest abundances in MDS-EB, and CEP55 was identified with the highest comparative abundance in the bone marrow plasma of MDS-RS patients. As the next phase of the research, gene expression validation of *CEP55*, *MSN*, *TLN1*, and *UBC* was carried out by qPCR in bone marrow mononuclear cells from 45 MDS patients, including 15 MDS-RS and 23 MDS-EB patients, five with multilineage dysplasia, one with single lineage dysplasia, and one with MDS secondary to therapy (**Table 2**). MDS patients were evaluated according to the IPSS-R (5). Four MDS-RS and three MDS-EB patients listed in Study 1 were included in Study 2.

**Table 2:** Summary of clinical and prognostic characteristics of patients with myelodysplastic syndrome (MDS).

Case	Gender	Age	Hb (g/dL)	ANC (/mm³)	Platelets (/mm³)	Karyotype	WHO 2016	IPSS-R
1	F	70	8.07	2294	449000	NO METAPHASE	MDS-RS	–
2	M	62	7.20	275	17000	47,XY,+8[6]/47,XY,del(7)(q32),+8[7]/46,XY[2]	MDS-EB	HIGH
3	M	82	7.63	3599	338000	46,XX[20]	MDS-RS	LOW
4	M	68	5.61	289	24000	NO METAPHASE	MDS-EB	–
5	F	82	5.87	2180	267000	46,XX[5]	MDS-RS	VERY LOW
6	M	74	8.60	3981	177600	46,XY[6]	MDS-RS	LOW
7	F	44	6.76	1887	9982	46,XX[20]	MDS-EB	HIGH
8	M	73	7.10	460	26000	NO METAPHASE	MDS-EB	–
9	F	76	8.70	3391	382000	46,XX[15]	MDS-RS	LOW
10	M	89	7.10	1600	104000	46,XY,t[5;19)(q13.2;q13.4)[3]/46,XY,t[5;19)(q13.2;q13.4),t(8,21)(q21.3;q22.12)[3]/46,XY,del(X)(q21),t(5;19)(q13.2;q13.4),t(8;21)(q21.3;q22.12)[5]/46,XY[9]	MDS-EB	VERY HIGH
11	M	58	7.80	2300	362000	46,XY,del(5)(q32)[3]/46,XY,del(5)(q32),del(7)(q36)[3]/46,XY,-5,+mar[9]/46,XY[7]	MDS-EB	HIGH
12	M	55	4.60	496	81000	45,XY,-7[15]/46,XY,-7,+mar[5]	MDS-EB	HIGH
13	F	42	10.50	2072	25000	46,XX[20]	MDS-EB	HIGH
14	M	84	3.90	2940	68000	46,XY[20]	MDS-EB	HIGH
15	F	79	9.90	1296	30000	46,XX[12]	MDS-EB	HIGH
16	M	75	8.30	957	21000	92, XXYY[4]	MDS-EB	HIGH
17	M	55	6.10	4460	40000	45,X,-Y[15]/45,X,-Y,del(5)(q32)[3]/46,XY[2]	MDS-EB	HIGH
18	F	81	11.90	744	57000	46,XY[4]	MDS-EB	HIGH
19	F	93	9.50	860	47000	46,XX,+8[12]/46,XX[8]	MDS-EB	HIGH
20	F	80	8.80	5461	21000	46,XX[20]	MDS-RS	LOW
21	F	77	12.00	1099	143000	46,XX[20]	MDS-EB	HIGH
22	F	82	6.90	1585	193000	47,XX,+8[9]/47,XX,+8,del(20)(q12)[5]/46,XX[6]	MDS-RS	INTERMEDIATE
23	M	79	9.90	7400	169000	46,XY[20]	MDS-EB	INTERMEDIATE
24	F	42	10.80	2079	147000	NO METAPHASE	MDS-RS	–
25	M	91	7.70	4753	203000	46,X-Y[4]/46,XY[16]	MDS-RS	LOW
26	M	58	8.50	494	300000	46,XY,del(5)(?q15q33)[8]/46,XY[12]	MDS-RS	LOW
27	M	79	6.70	4752	16000	NO METAPHASE	MDS-EB	–
28	F	72	6.50	3619	18040	NO METAPHASE	MDS-EB	–
29	F	83	9.60	1870	82000	46,XX[20]	MDS-EB	HIGH
30	F	59	9.60	3080	326000	46,XX[10]	MDS-RS	LOW
31	M	60	6.12	3643	51830	47,XY,+15[10]/46,XY[10]	MDS-RS	LOW
32	F	44	6.76	1887	9982	46,XX[20]	MDS-EB	HIGH
33	F	46	4.80	2767	556100	46,XX[20]	MDS-MLD	LOW
34	F	70	6.80	1357	23000	48,XX,del(9)(q22),r(10)(p15q26.3),+16,+18[15]/46,XX[5]	t-MDS	HIGH
35	F	56	5.2	864	28000	46,XX[20]	MDS-EB	HIGH
36	M	52	7.0	3203	6000	46,XY,del(7)(q31)[3]/45,XY,-5,del(7)(q31)[5]/46,XY,del(5)(q15),del(7)(q31)[8]46,XY,del(7)(q31),add(11)(q24)[4]/46,XY,del(5)(q15),del(7)(q31),add(11)(q24)/46,XY[5]	MDS-EB	HIGH
37	F	76	9.30	4120	234000	46,XX[20]	MDS-RS	HIGH
38	F	64	10.90	1232	20700	46 XX [10]	MDS-MLD	LOW
39	M	70	10.20	947	117400	46,XY[20]	MDS-RS	LOW
40	F	48	11.60	751	57000	46,XX[4]	MDS-SLD	LOW
41	F	76	9.90	308	90590	47,XX,+22[4]/46,XX[16]	MDS-MLD	LOW
42	M	44	15.80	852	218000	46,XY,del(5)(q15q33), del(17)(p11.2)[7]/46,XY[13]	MDS-MLD	LOW
43	F	47	8.60	2920	30000	47,XX,+6[3]/46,XX[17]	MDS-MLD	LOW
44	M	71	5.10	813	202000	46,XY[20]	MDS-EB	HIGH
45	M	92	9.20	2204	182400	46,XY,del(5)(q15q33)[7]/46,XY[13]	MDS-RS	LOW

ANC, Absolut Neutrophil Count; F, Female; Hb, Hemoglobin; IPSS-R, Revised International Prognostic Score System; M, Male; MDS-EB, MDS with excess blasts (n = 23); MDS-MLD, MDS with multilineage dysplasia (n = 5); MDS-SLD, MDS with single lineage dysplasia (n = 1); t-MDS, MDS secondary to therapy (n = 1); MDS-RS MDS with ring sideroblasts (n = 15).

##### 2.2.2.1 Total RNA Extraction and Quantitative Real-Time PCR

Bone marrow was obtained by sternal aspiration of all patients, as described in Study 1. After centrifugation and removal of plasma, mononuclear cells were separated after lysis of red blood cells and subjected to total RNA extractions using Trizol Reagent™ (Invitrogen, Carlsbad, CA, USA), followed by RNA quantification (NanoDrop 2000c; Thermo Scientific, USA). Complementary DNA (cDNA) was obtained using a High-Capacity cDNA Reverse Transcription kit (Applied Biosystems, Foster City, CA, USA) according to the manufacturer’s recommendations. Quantitative real-time PCR (qPCR) reactions were performed using the TaqMan methodology (Applied Biosystems, USA) on 7500 Fast System^®^ (Applied Biosystems, USA). The custom TaqMan Gene Expression Assays were used for *CEP55* (Hs01070181_m1), *MSN* (Hs00792607_mH), *TLN1* (Hs00196775_m1), and *UBC* (Hs00824723_m1) and the reference gene *B2M* (beta-2-microglobulin, Hs99999907_m1). *B2M* has been previously validated as a stable reference in gene expression analysis of bone marrow mononuclear cells from MDS patients (38). PCRs were performed using 1× TaqMan^®^ Universal Master Mix II, with UNG^®^ (Applied Biosystems, USA), 1× μl of each probe, and 2× μl of cDNA. The following cycling conditions were performed: initial denaturation at 95°C for 15 min followed by 40 cycles consisting of 95°C for 15 s and 60°C for 1 min. Each sample was performed in duplicate, and the expression ratios were calculated using the 2^−ΔCq^ method (39).

### 2.3 Statistical Analysis

Normality of mRNA data distribution was evaluated by the Shapiro–Wilk test and Student’s t-test or one-way ANOVA with Tukey/Games–Howell *post-hoc* test used when normality was detected. The variance homogeneity for all variables analysis was evaluated by Levene’s test. Statistical analyses were performed using SPSS 21.0 (SPSS Inc., Chicaog, IL, USA) and GraphPad Prism 8 (GraphPad Software, La Jolla, CA, USA) software.

### 2.4 *In Silico* Analyses of Protein–Protein Interactions, MiRNA-Linked Networks, and CD34+ Gene Expression

Networks of TLN1 and CEP55 proteins were analyzed using String platform version 11.5 (https://string-db.org/ (31, 40)). Protein physical interactions, network type, network edges, and active interaction sources were based on String default settings, using the *Homo sapiens* National Center for Biotechnology Information (NCBI) database. The number of interactions was limited to 10, considering 0.900 as the minimum interaction score. Interactions of CEP55, MSN, TLN1, and UBC were evaluated with the GeneMANIA platform (www.genemania.org) (41). Protein physical interactions, co-expression, predicted networks, co-localization, pathways, genetic interactions, and shared protein domains were analyzed using the default GeneMANIA approach (automatically selected weighting method) and the *Homo sapiens* gene nomenclature.

The potential regulation of *TLN1* and *CEP55* genes by miRNAs was analyzed using the miRNet 2.0 database (https://www.mirnet.ca) (42). The network was built considering a miRNA database for humans and bone marrow as a specific tissue and using a 1.0 degree cutoff of a node. The degree represents the number of connections, and nodes with a higher degree are important hubs in a network. Moreover, functional enrichment of all miRNAs was built, with highlights for main biological functions.

We performed *in silico* evaluation of *CEP55*, *TLN1*, and *MSN* gene expression in CD34+ cell markers from MDS patients presented in the Gene Expression Omnibus (GEO) data bank (https://www.ncbi.nlm.nih.gov/geo/query/acc.cgi?acc=GSE58831), as described by Gerstung et al. (43) This study evaluated gene expression from 159 patients with MDS patients and 17 healthy controls from bone marrow samples, and microarray gene expression was performed using the Affymetrix GeneChip Human Genome U133 Plus 2.0 arrays.

## 3 Results

### 3.1 Study 1: Proteome of the Bone Marrow Plasma of Myelodysplastic Syndrome Patients

**Figure 2:**
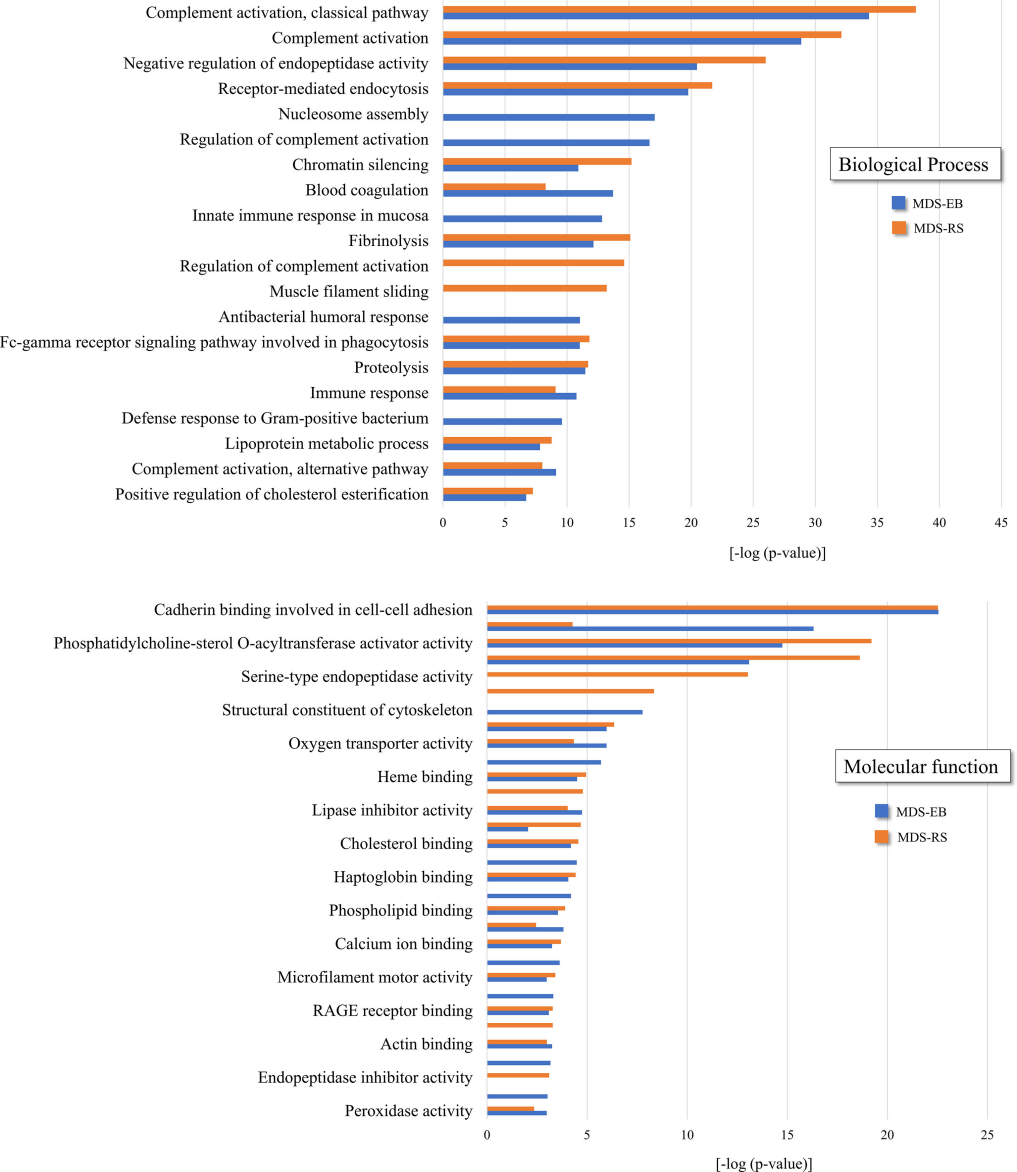
Functional clusters associated with bone marrow plasma proteins of patients diagnosed with MDS-RS and MDS-EB, according to DAVID Functional Annotation Bioinformatics Analysis (https://david.ncifcrf.gov). UniProt accession numbers were uploaded to DAVID platform, and clusters were defined by enrichment scores and p-values [−log (p-value)]. Information shown in the present figure is derived from the complete list of functional clusters presented in **Supplementary Table 1**.

As determined by LC-MS/MS, 1,194 proteins were identified in the bone marrow plasma of MDS patients, with 320 and 490 proteins unique to MDS-RS and MDS-EB samples, respectively, and 384 proteins common to both MDS subtypes (**Supplementary Table 1A**). UniProt accession codes of the 874 proteins identified in the bone marrow plasma of patients diagnosed with MDS-EB were uploaded to the DAVID platform and matched to 310 gene codes, grouped in 54 clusters. In contrast, 704 proteins identified in the bone marrow plasma of MDS-RS patients matched 251 gene codes, organized in 64 clusters. Clusters with the highest enrichment scores associated with both MDS-EB and MDS-RS included biological processes such as complement activation, negative regulation of endopeptidase activity, and receptor-mediated endocytosis. The most enriched molecular functions were defined as cadherin binding involved in cell–cell adhesion, actin binding, phosphatidylcholine binding, and conversion and serine-type endopeptidase activity, among others (**Figure 2**; **Supplementary Tables 1B, C**).

#### 3.1.1 Differentially Abundant Proteins in the Bone Marrow Plasma of Patients With Myelodysplastic Syndrome With Ring Sideroblasts and Myelodysplastic Syndrome With Excess Blasts

**Figure 3:**
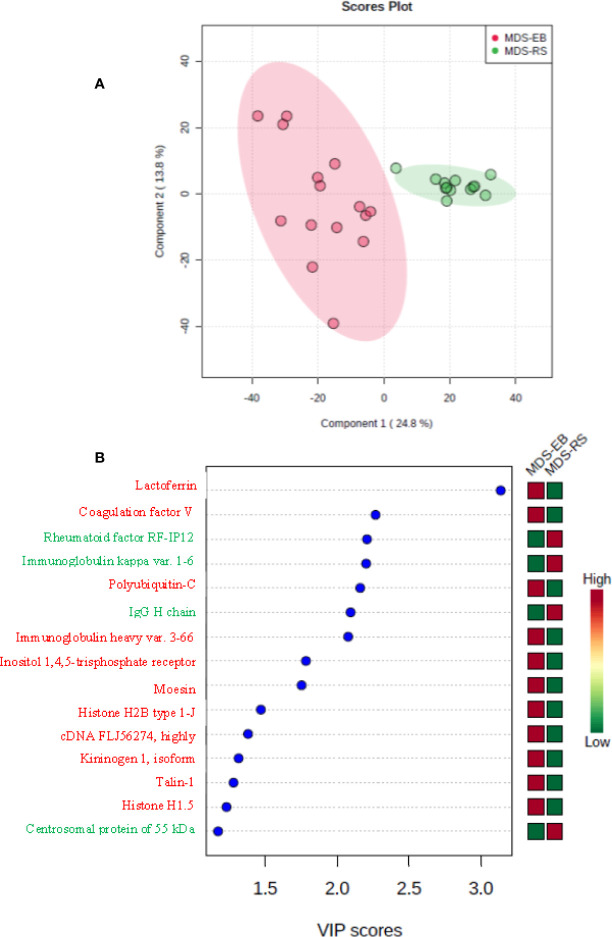
**(A)** Partial least square discriminant analysis (PLS-DA) of protein abundances in the bone marrow plasma of patients with myelodysplastic syndrome with ring sideroblasts (MDS-RS) and with excess blasts (MDS-EB). Explained variances of components are shown in brackets. **(B)** Variable importance in projection (VIP) scores associated with bone marrow plasma proteins, as identified by PLS-DA. Colored boxes on the right indicate the relative abundances of proteins in each MDS subtype.

Based on the label-free mass spectrometry and data analysis by Progenesis QI software, there were 73 differentially abundant proteins (p < 0.05) in the bone marrow plasma of MDS-RS and MDS-EB patients (**Supplementary Table 2**). The partial least square discriminant analysis confirmed a pronounced contrast in the protein profiles of bone marrow plasma from MDS patients (**Figure 3A**). According to VIP scores, proteins identified as Lactoferrin, Coagulation factor V, Polyubiquitin-C, Immunoglobulin heavy variable 3-66, Inositol 1,4,5-trisphosphate receptor, MSN, Histone H2B type 1-J, cDNA FLJ56274, Kininogen 1 isoform, Talin-1, and Histone H1.5 made the most meaningful contributions to characterize the bone marrow plasma of MDS-EB patients. In contrast, Rheumatoid factor RF-IP12, Immunoglobulin kappa var. 1-6 IgG H chain, and CEP55 defined the major representation of bone marrow plasma from MDS-RS patients (**Figure 3B**; **Table 3**). Considering fold-change values, Polyubiquitin-C (UBC), MSN, and Talin-1 (TLN1) showed the highest relative abundances in MDS-EB, and CEP55 was the major protein of the bone marrow plasma from MDS-RS patients (**Table 3**; **Supplementary Table 2**).

**Table 3:** Proteins of the bone marrow plasma with the highest VIP scores and different abundances in patients with myelodysplastic syndrome with ring sideroblasts (MDS-RS) and patients diagnosed with myelodysplastic syndrome with excess blasts (MDS-EB).

Accession number	Protein description	Higher abundance in:	Max. fold change	Confidence score	Anova (p)
Q53EZ4	Centrosomal protein of 55 kDa GN=CEP55	MDS-RS	Infinity	24.72	0.003314
P26038	Moesin GN=MSN	MDS-EB	2713.22	71.69	0.000174
F5GZ39	Polyubiquitin-C (Fragment) GN=UB	MDS-EB	2219.84	46.85	0.004344
Q9Y490	Talin-1 GN=TLN	MDS-EB	294.77	268.42	0.022615
P06899	Histone H2B type 1-J GN=HIST1H2B	MDS-EB	138.86	47.59	0.013181
P16401	Histone H1.5 GN=HIST1H1B	MDS-EB	135.53	90.01	0.009784
P12259	Coagulation factor V GN=F	MDS-EB	104.46	25.06	0.020701
W8QEY1	Lactoferrin GN=FTF	MDS-EB	68.65	59.03	0.002933
B4E022	cDNA FLJ56274. highly similar to Transketolase (EC 2.2.1.1)	MDS-EB	68.38	203.17	0.001416
Q59ES2	Inositol 1,4,5-trisphosphate receptor type 3 variant (Fragment)	MDS-EB	56.65	30.24	0.008421
A2J1M8	Rheumatoid factor RF-IP12 (Fragment)	MDS-RS	25.93	40.95	4.88E-08
S6AWF0	IgG H chain	MDS-RS	12.07	52.97	1.94E-06
B4E1C2	Kininogen 1. isoform CRA_b OS=Homo sapiens GN=KNG1 PE=2 SV=1	MDS-EB	11.23	32.27	0.009069
A0A0C4DH72	Immunoglobulin kappa variable 1-6 GN=IGKV1-6	MDS-EB	5.89	31.18	0.001043
A0A0C4DH42	Immunoglobulin heavy variable 3-66 GN=IGHV3-66	MDS-EB	2.57	27.42	0.021628

Proteins were identified by label-free mass spectrometry and data, analyzed by Progenesis QI software. Listed proteins as depicted in **Figure 3B**.

### 3.2 Study 2: Gene Expression in Mononuclear Cells of Myelodysplastic Syndrome Patients

**Figure 4:**
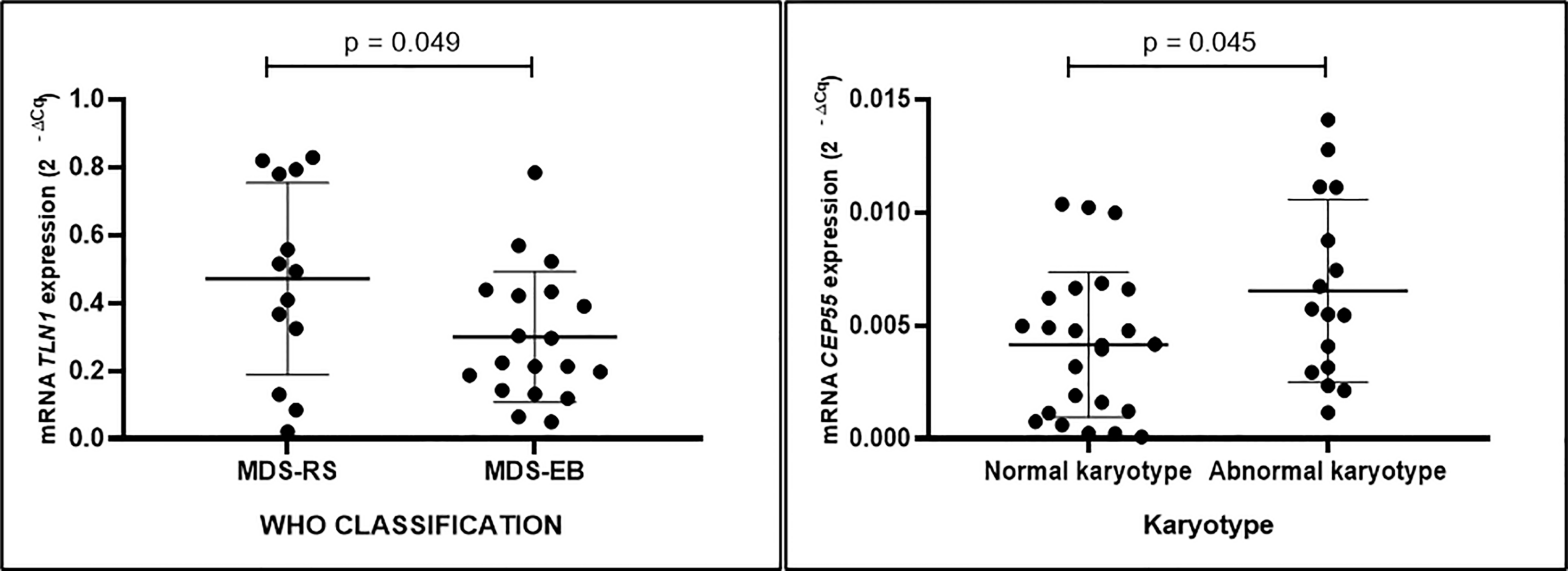
Quantitative data of *TLN1* and *CEP55* expression (2^−ΔCq^) based on qPCR analysis of total RNA extracted from bone marrow mononuclear cells of patients with myelodysplastic syndrome with ring sideroblasts (MDS-RS) and with excess blasts (MDS-EB) and patients with normal and abnormal karyotypes, as listed in **Table 2**.

Expression of *CEP55*, *MSN*, and *UBC* was similar in mononuclear cells from individuals with MDS-RS and MDS-EB (p > 0.05). *TLN1* gene expression, however, was greater in mononuclear cells of MDS-RS than in mononuclear cells of MDS-EB patients (p = 0.049). Relative quantities of *MSN*, *UBC*, and *TLN1* transcripts were similar (p > 0.05) in mononuclear cells of individuals with normal and abnormal karyotypes, irrespective of the MDS subtype. However, *CEP55* gene expression was greater in MDS patients with abnormal karyotypes in comparison with patients with normal karyotypes (p = 0.045; **Figure 4**; **Supplementary Table 3**).

#### 3.2.1 Protein Networks, MiRNA–Gene Interactions, and CD34+ Cell Gene Expression

**Figure 5:**
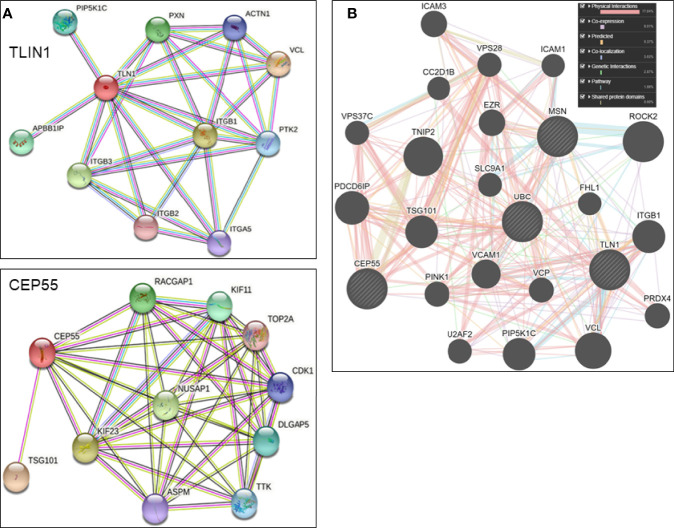
**(A)** Individual *in silico* networks associated with TLN1 and CEP55 according to String version 11.5 default settings (https://string-db), using *Homo sapiens* NCBI database. **(B)** Combined *in silico* networks of CEP55, MSN, TLN1, and UBC as determined by the default approach of GeneMANIA platform (www.genemania.org).

As determined by *in silico* models, the most significant (score ≥ 0.977) components of TLN1 interactome included vinculin, actin filament (F-actin)-binding protein, integrin beta-1, 2 and 3, and alpha-5, paxillin, amyloid beta A4 precursor protein-binding family B member 1-interacting protein, phosphatidylinositol 4-phosphate 5-kinase type-1 gamma, focal adhesion kinase 1, and alpha-actinin-1. The array of CEP55 interactions (score ≥ 0.969) included tumor susceptibility gene 101 protein, kinesin-like protein KIF23, nucleolar and spindle associated protein 1, Rac GTPase-activating protein 1, kinesin-like protein KIF11, disks large-associated protein 5, protein kinase TTK, cyclin-dependent kinase 1, abnormal spindle-like microcephaly-associated protein, and a DNA topoisomerase (**Figure 5A**). *In silico* analysis using the GeneMANIA platform indicated that CEP55, MSN, TLN1, and UBC shared pathways, domains, and physical interactions (**Figure 5B**).

**Figure 6:**
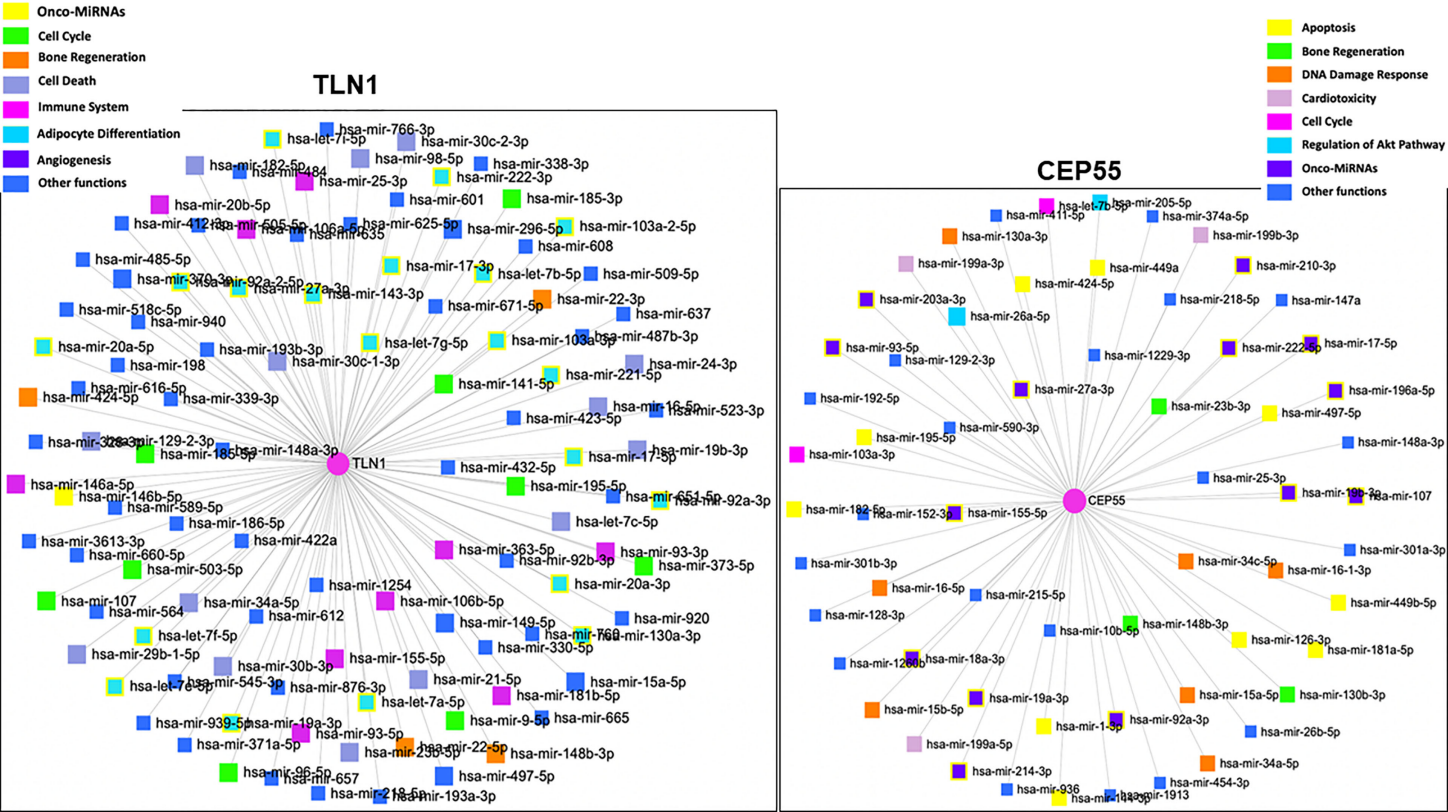
Network and gene set enrichment analysis of miRNAs associated with the regulation of human *TLN1* and *CEP55* according to the highest p-values obtained from miRNet database (https://www.mirnet.ca).

**Figure 7:**
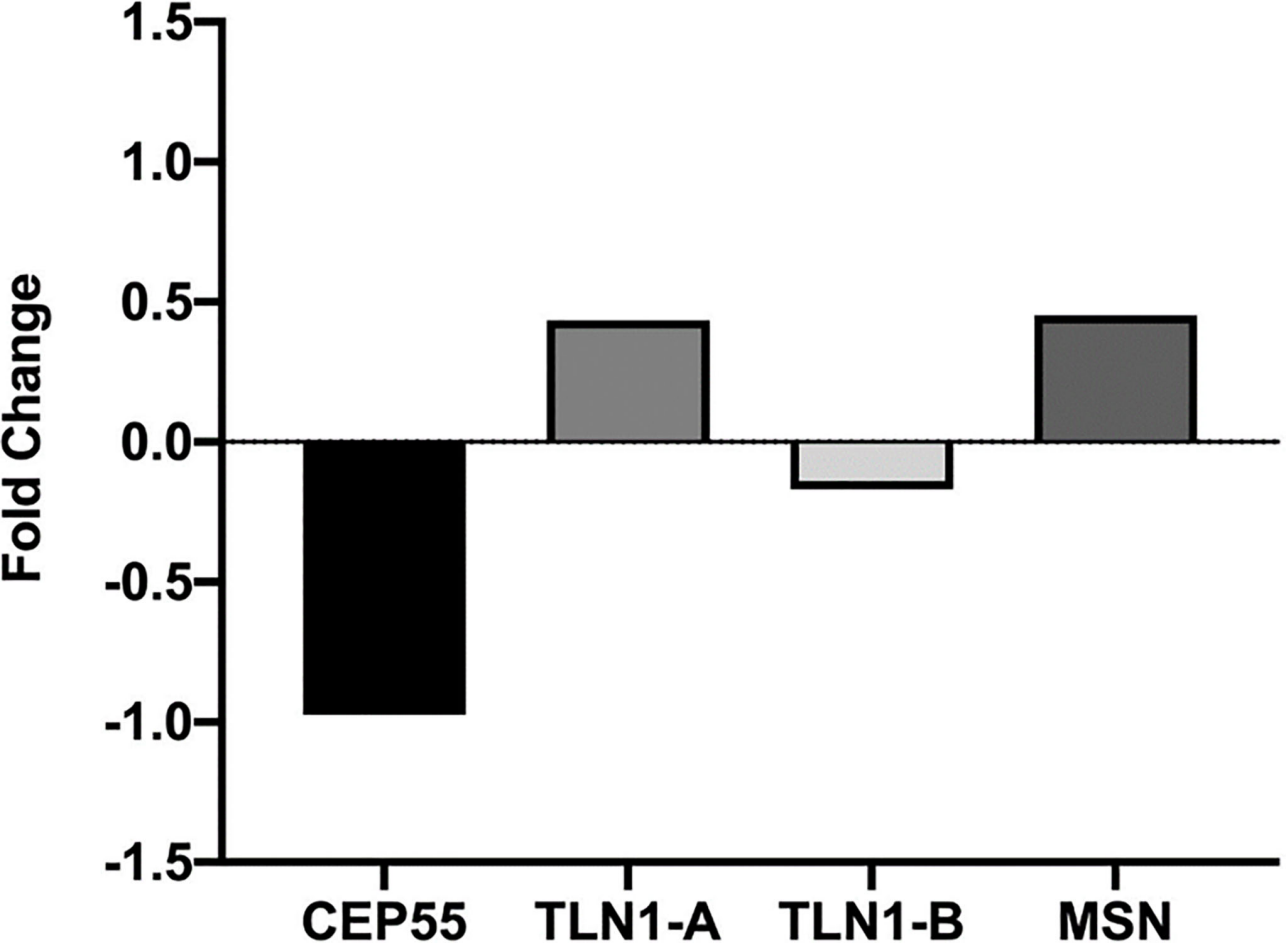
Relative *CEP55*, *TLN1*, and *MSN* expression in CD34+ cell markers from MDS patients presented in the Gene Expression Omnibus data bank (https://www.ncbi.nlm.nih.gov/geo/query/acc.cgi?acc=GSE58831). Analysis was conducted *in silico* based on bone marrow samples of 159 MDS patients and 17 healthy controls, and microarray gene expression was performed using the Affymetrix GeneChip Human Genome U133 Plus 2.0 arrays.

Based on the analysis conducted with the miRNet 2.0 database, *TLN1* is regulated by onco-miRNAs and other miRNAs associated with cell cycle, bone regeneration, cell death, immune system, angiogenesis, and hematopoiesis, among others (**Figure 6**). *CEP55* gene was regulated by miRNAs associated with bone regeneration, DNA damage, cardiotoxicity, cell cycle, regulation of Akt pathway, and onco-miRNAs, among others (**Figure 6**; **Supplementary Table 4**). *In silico* analysis of CD34+ cell gene expression showed that *CPE55* expression was reduced while both *TLN1* and *MSN* were increased in MDS patients compared to healthy controls (**Figure 7**; **Supplementary Tables 1D, E**).

## 4 Discussion

Myelodysplastic syndromes are clinically heterogeneous and characterized by dysplasia of bone marrow cells, peripheral blood cytopenia, bone marrow insufficiency, disrupted hematopoiesis, and anemia, among other factors (1, 2–4). Molecular determinants of MDS pathobiology are complex and involve altered genomic and epigenetic factors, protein synthesis and structure, intracellular signaling, and mechanisms that control cell communication and fate. Therefore, our current results advance our knowledge about the molecular landscapes of MDS.

Mass spectrometry coupled with nano-high-performance liquid chromatography (nano-HPLC) allowed the identification of 1,194 proteins in the bone marrow plasma of MDS patients. Gene ontology biological processes and molecular functions basically followed the same pattern for BM plasma proteins of MDS-RS and MDS-EB patients, including complement activation and immune-related events. In fact, chronic inflammatory diseases associated with activated innate immune signaling pathways usually precede MDS, creating the concept that this event in hematopoietic stem cells (HSC) relates to MDS etiology. Immune response-related genes in HSC are overexpressed in more than 50% of MDS patients (44), and our analysis demonstrates that response to stimulus and immune system process were major biological processes associated with the BM plasma proteome of patients with MDS-RS and MDS-EB. Functional clusters associated with the BM plasma proteome included, as expected, immune mechanisms, endocytosis, nucleosome assembly, chromatin silencing, blood coagulation and fibrinolysis, cytolysis, and cell–cell adhesion, among others. There is an important interplay between chronic immune stimulation and MDS pathogenesis. In agreement with this concept, a study conducted by Pellagatti et al. (45) reported upregulation of interferon-stimulated genes in CD34+ cells obtained from the bone marrow of MDS patients. There is also robust evidence of immune deregulation in MDS due to the presence of autoreactive T-cell clones and that interferon γ had meaningful inhibitory effects on hematopoietic stem cells. We have previously detected, in a study with 111 MDS patients (46), that interferon regulatory genes are upregulated and linked to MDS pathogenesis, confirming the fundamental link between genomic instability and immune activation.

Considering the scenario with 1,194 proteins identified in the bone marrow plasma, univariate and multivariate statistical approaches grouped 15 proteins that most defined MDS-RS and MDS-EB subtypes. Among this select group, TLN1, MSN, and UBC had the greatest abundances in the bone marrow plasma of MDS-EB, and CEP55 showed the highest abundance in MDS-RS patients. CEP55 is a major player in cytokinesis, participates in PI3K/AKT signaling pathway, and is associated with tumor progression (47). MSN, in turn, connects actin filaments with the plasma membrane and contributes to cell shape, membrane transport, signal transduction, T- and B-cell homeostasis, and macrophage phagocytosis. Experimental evidence support UBC’s roles in autophagy and signaling in cancer cells (48) and attachment of ubiquitin types to other proteins modulate DNA repair, cell-cycle communication, lysosomal and protein degradation, and kinase activation, among other events (49). As a cytoskeleton molecule, TLN1 connects integrin domains to actin and intermediate filaments, helping cell–cell and cell–extracellular matrix (ECM) attachment, cell migration, and signal transduction (50, 51). *In silico* analysis based on the GeneMANIA platform showed pathways, domains, and physical interactions shared by CEP55, MSN, TLN1, and UBC, indicating that the actual work of these proteins certainly involves more complex mechanisms than those foreseeing when only their individual roles are analyzed.

Proteomics data gathered in Study 1 and the well-known bio tasks of CEP55, MNS, UBC, and TLN1 prompted us to investigate the expression of these genes in a follow-up study with 45 patients. In this case, *TLN1* was overexpressed in mononuclear cells from MDS-RS when compared to MDS-EB patients. TLN1 contains alpha-helices and post-translational modification sites (https://alphafold.ebi.ac.uk/entry/Q9Y490), with actin- and integrin-binding domains (50, 52, 53). TLN1 is a player in mechanotransduction and filopodia function (54, 55), and talin dysregulation results in pathological phenotypes with altered cell spreading, migration, and survival (56, 57). Talin is associated with enhanced cell proliferation (common phenomena of blasts in MDS-EB), anoikis resistance and metastasis (57, 58), hematologic disorders (54), and platelet and neutrophil activation (59). Talin-integrin interdependence affects endothelium linkage, migration of platelets and neutrophils (59, 60) and leukocyte function (61), and *TLN1* knockout mouse embryos have defective angiogenesis (62). From the perspective of clinical practice, we know that MDS-EB shows a high risk of AML transformation due to an increase in proliferation and survival of blasts. Thus, functional studies are needed to evaluate if TLN1 expression is related to AML transformation and, if so, this information will generate markers of MDS pathogenesis and possible targets of treatment.

Why, in our research, TLN1 was more abundant in the bone marrow plasma of MDS-EB and *TLN1* gene was prevalent in mononuclear cells of MDS-RS patients is not clearly understood. Bone marrow plasma obviously contains molecules synthesized by BM cells and components from blood, suggesting that the content of BM plasma does not entirely reflect what comes from mononuclear cells. Moreover, mRNA and protein quantities in a given biological entity can be unrelated (63–66), and cell lysis and/or apoptosis may have occurred during the collection and separation of BM plasma. Another possible explanation is that MDS-RS cases present serious problems of splice machinery (mutation of *SF3B1*), leading to disrupted mRNA translation, with increases in some mRNAs without proportional changes in protein synthesis. In this regard, comparisons between protein abundances in BM plasma and mRNA quantities in mononuclear cells are not straightforward because two levels of molecular information were actually generated in our studies, i.e., proteomics of BM plasma and qPCR in mononuclear cells. Thus, these technical approaches provide complementary and not conflicting pieces of information. High TLN1 abundance in the BM plasma of patients with a high-risk MDS subtype (MDS-EB) may relate to TLN1-mediated activation of FAK/AKT signaling and anoikis resistance (67). Low *TLN1* gene expression detected in mononuclear cells of MDS-EB patients could be the result of impaired talin/integrin complex.

*In silico* analyses outline TLN1 interactions with proteins that regulate cell–cell and cell–ECM adhesion, cell migration, apoptosis, and intracellular signals, such as vinculin, integrins, and A4 precursor protein-binding family B member 1-interacting protein (APBB1IP). Vinculin is downregulated in mononuclear cells of MDS patients (21), and APBB1IP is a RAS-related protein acting in high cell proliferation and AML transformation in MDS-EB cases (68). *In silico* models also identified that onco-miRNAs and miRNAs linked to cell cycle control and death, immune system, angiogenesis, and hematopoiesis are capable of regulating *TLN1* expression. Some of those miRNAs, such as miR-17-3p and miR-17-5p, are upregulated in the BM of patients with MDS-RS, refractory cytopenia with multilineage dysplasia (RCMD), and RCMD with ringed sideroblasts, according to a recent review (69) and other studies (70–72). Other miRNAs, such as miR-27a-3p and miR-16-5p, were reduced in the plasma of patients with refractory cytopenia with unilineage dysplasia, refractory anemia with ringed sideroblasts, RCMD, MDS-EB1, and MDS-EB2. Hematopoiesis-related miR-34a-5p and miR-146b-5p were increased in plasma and extracellular vesicles of MDS patients with multilineage dysplasia, MDS-RS, MDS with isolated del(5q), MDS-EB 1, and MDS-EB 2. Given its functional properties and association with cancer processes (54, 73), TLN1 is indeed a potential drug for targeted therapy (74), but more investigation is needed to decipher how drugs should be developed to tackle protein action and/or *TLN1* expression. Considering that hematopoietic stem and progenitor cells (HSPCs) are important for MDS prognosis and treatment, we performed an *in silico* analysis of CD34+ gene expression and verified that *CEP55* was reduced and both *TLN1* and *MSN* expressions were increased in the bone marrow of MDS patients without any stratification.

As defined in Study 1, CEP55 was more abundant in the bone marrow plasma of MDS-RS patients. Remarkably, we observed in Study 2 that increased *CEP55* expression in mononuclear cells was linked to chromosomal abnormality in MDS patients, regardless of MDS subtypes. CEP55 has domains (https://alphafold.ebi.ac.uk/entry/Q53EZ4) involved in abscission, midbody localization (75), dimerization (76–78), and a ubiquitin-binding domain required for CEP55 action on cytokinesis (75, 77). CEP55 influences aneuploid cells during perturbed mitosis (79), and high CEP55 expression in murine models facilitates exit from mitotic arrest, resulting in aneuploidy and resistance to anti-mitotic drugs. CEP55-depleted organisms have increased multinucleated cells and/or cells arrested at the midbody stage (80). Based on *in silico* modeling, CEP55 interacts with components of the ESCRT-I complex, a regulator of vesicular transport, cell growth, and differentiation; a microtubule-associated protein; a regulator of Rac GTPase activity and cell cycle during carcinogenesis; and cyclin-dependent kinases. This scenario reflects the multidimensional attributes of CEP55. Similar to *TLN1*, the expression of *CEP55* is potentially regulated by oncogenes and miRNAs linked to DNA damage response, cell cycle, and AKT pathway. Several genes that define normal blood cells are regulated by miRNAs, and MDS prognosis is linked to certain miRNAs of the bone marrow, peripheral blood, and mononuclear cells (69). In fact, some *CEP55*-controlling miRNAs identified in our *in silico* model have been listed as differentially expressed in other patients with MDS (70, 71, 81, 82).

Chromosomal abnormalities occur in up to 50% of MDS cases, mostly caused by problems in the spindle assembly checkpoint (SAC), which controls mitotic progression, chromosome alignment, and segregation. SACs delay anaphase until accurate kinetochore-microtubule attachment at metaphase and inhibition of SAC prolongs mitotic arrest. The majority of antimitotic chemotherapies alter microtubule dynamics, but cancer cells can bypass mitotic arrest and prematurely exit mitosis. High *CEP55* expression is a marker of chromosomal alterations, aneuploidy, and poor prognosis of cancer patients (79, 83, 84) and promotes tumorigenesis in transgenic mice (85) as well. Clinical practice indicates that overall survival reaches 3 years in MDS patients with normal karyotypes, while cases with complex karyotypes show a 6-month survival. As CEP55 consistently relates to mitotic slippage in certain types of cancers, we urgently need to study the effects of *CEP55* reduced expression and if CEP55 plays a central role in the genesis of cytogenetic abnormalities.

In conclusion, proteomic and gene expression approaches were used to unfold some of the molecular signatures related to MDSs. We recognize that our study has limitations, as we did not evaluate other MDS subtypes such as MDS with multilineage dysplasia, MDS secondary to therapy, and hypoplastic MDS. Also, gene expression was not analyzed in specific HSPC such as CD34+ cells, a marker for prognosis and treatment of MDS. To our knowledge, however, this is the first evidence of altered TLN1 and CEP55 in both cellular and non-cellular bone marrow compartments of patients with low- and high-risk MDS and with normal vs. abnormal karyotypes. The current diagnostic criteria and classification for MDS include bone marrow chromosomal alterations, blast count, and cytopenia, which are important to be analyzed for the prognosis of untreated patients. Given the complex and multifactorial characteristics of MDS, a deep molecular investigation of altered pathways provides a better understanding of the disease prognosis, its evolution to AML, and resistance to treatment. The novel potential markers CEP55 and TLN1 bring insights into the molecular signatures of MDS, setting the foundation for further investigations with stratified patients and HSPC and helping in the MDS prognosis and targeted therapy.

## Data Availability Statement

The datasets presented in this study can be found in online repositories. The names of the repository/repositories and accession number(s) can be found below: https://massive.ucsd.edu/ProteoSAFe/static/massive.jsp MassIVE ID=MSV000088457.

## Ethics Statement

The studies involving human participants were reviewed and approved by the Ethics Committee of the Federal University of Ceará, UFC (#69320817.7.0000.5054). The patients/participants provided their written informed consent to participate in this study. Written informed consent was obtained from the individual(s) for the publication of any potentially identifiable images or data included in this article.

## Author Contributions

AAM, CLMF, AMAM, MOM-F, CP, and RFP designed the study and the overall organization of the article. RTGO and RFP collected the bone marrow samples and analyzed the karyotypes. MJBB, AMAM, WF, and RMO prepared the samples and carried out the proteomic analysis. MVS, WF, and CAOR provided the expertise and technical and financial support for the mass spectrometry, data search, and Progenesis analysis. DPB, RTGO, and CLMF did the RNA isolation from mononuclear cells and conducted qPCR and gene expression analysis. AGV, KMF, GGCC, and CRKP contributed to the bioinformatics analysis. MEAM, SMMM, MOM-F, AAM, CP, and RFP were responsible for the supervision of students and postdoctoral fellows and provided financial and institutional support, which was essential for the completion of the project. AAM, MJBB, CLMF, AMAM, CP, WF, RTGO and RFP contributed to the writing process. Final text editing was done by AAM, CLMF, AMAM, CP, and RFP. All authors listed have made a substantial, direct, and intellectual contribution to the work and approved it for publication.

## Funding

The present study was funded by the Ceará State Foundation for the Support of Scientific Development (FUNCAP) and The Brazilian Council for Science and Technology Development (CNPq) (grant # PR2-0101-00049.01.00/15). Additional support was provided by CNPq to CLMF (grant # 437037/2018-5), CP (grants # 440755/2018-2, 434821/2018-7, and 303102/2013-6), and AAM (grant # 313160/2017-1). The authors also appreciate the support of FUNCAP, CNPq, and The Brazilian Commission for Higher Education (CAPES) for graduate student and postdoctoral scholarships.

## Conflict of Interest

The authors declare that the research was conducted in the absence of any commercial or financial relationships that could be construed as a potential conflict of interest.

## Publisher’s Note

All claims expressed in this article are solely those of the authors and do not necessarily represent those of their affiliated organizations, or those of the publisher, the editors and the reviewers. Any product that may be evaluated in this article, or claim that may be made by its manufacturer, is not guaranteed or endorsed by the publisher.

## Supplementary Material

Supplementary Figure 1
**(A)** Uni-dimensional gel electrophoresis of bone marrow (BM) plasma proteins. Gels were made with 12.5% acrylamide and BM samples were obtained from a single MDS patient (not included in the present study). **(B)** Two-dimensional electrophoresis of BM plasma proteins (based on protocols we have previously described in detail (29)). Briefly, 13-cm strips (pH 3-10; GE Healthcare, USA) were rehydrated in a solution containing BM plasma proteins (300 µg) and subjected to isoelectric focusing. The second dimension was run as in **(A)**. All 1-D and 2-D gels were stained with Coomassie blue and scanned at 300 dpi (Image Scanner, GE Healthcare, USA). Gel # 1 refers to BM sample from a patient with MDS with single lineage dysplasia (MDS-SLD); gels # 3, 6 and 7 refer to samples of BM plasma from MDS-RS patients; gels # 2, 4 and 5 contain BM plasma proteins from MDS-EB patients.

Supplementary Table 1
**(A)** Proteins of the bone marrow plasma from patients (as listed in ) with myelodysplastic syndrome. Proteins were identified by label-free mass spectrometry and data, analyzed by Progenesis QI software. **(B)** and **(C)** Functional clusters associated with bone marrow plasma proteins of patients diagnosed with myelodysplastic syndrome with ring sideroblasts (MDS-RS) and with myelodysplastic syndrome with excess blasts (MDS-EB), according to DAVID Functional Annotation Bioinformatics Analysis (https://david.ncifcrf.gov). Uniprot accession numbers were uploaded to DAVID platform and clusters were defined by enrichment scores and p-values [-log (p-value)]. Data refer to patients listed in . **(D)** and **(E)** Quantitative data, statistical analysis and gene symbols associated with *in silico* analysis of *CEP55*, *TLN1* and *MSN* gene expression in CD34+ cell markers from MDS patients presented in the Gene Expression Omnibus (GEO) data bank (https://www.ncbi.nlm.nih.gov/geo/query/acc.cgi?acc=GSE58831). This study evaluated gene expression from 159 patients with MDS patients and 17 healthy controls from bone marrow samples and microarray gene expression was performed using the Affymetrix GeneChip Human Genome U133 Plus 2.0 arrays.
