# Supplementary figures and images for: Global Proteomics Analysis of Bone Marrow: Establishing Talin-1 and Centrosomal Protein of 55 kDa as Potential Molecular Signatures for Myelodysplastic Syndromes
Source: Front Oncol. 2022 Jun 22;12:833068. doi: 10.3389/fonc.2022.833068 (PMC9257025; doi:10.3389/fonc.2022.833068)

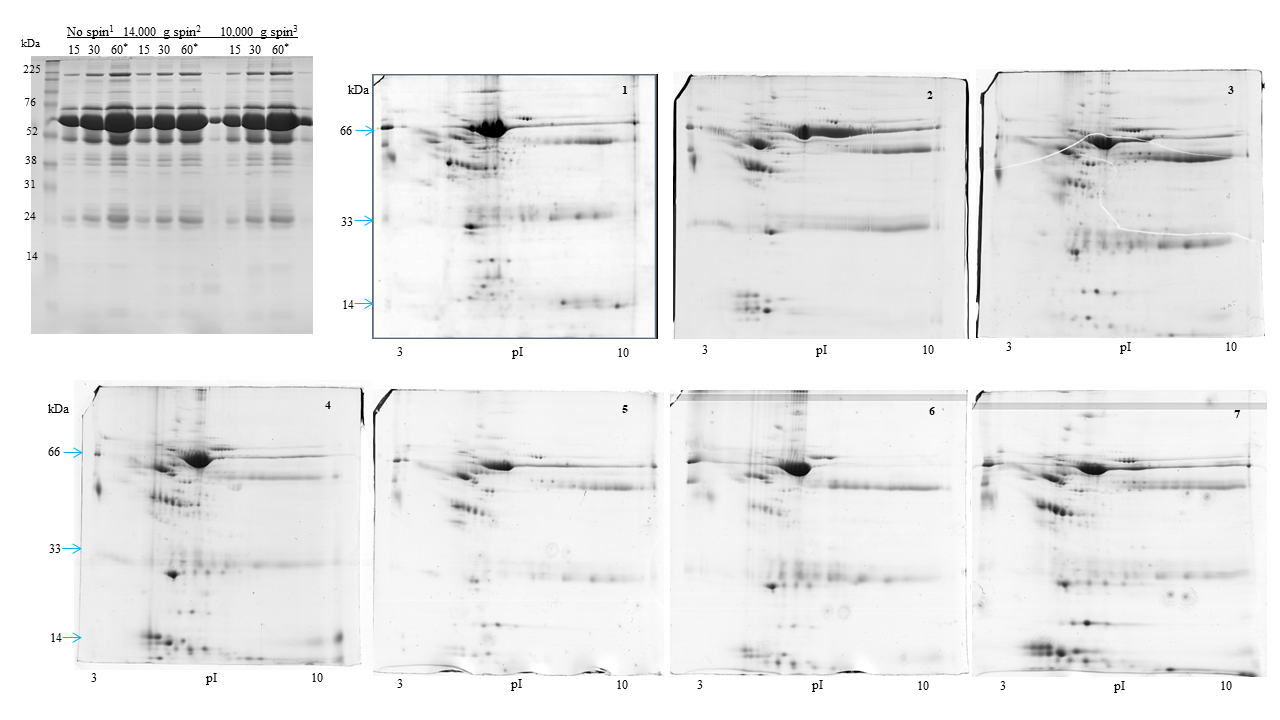

Supplement: Supplementary Figure 1 — (A) Uni-dimensional gel electrophoresis of bone marrow (BM) plasma proteins. Gels were made with 12.5% acrylamide and BM samples were obtained from a single MDS patient (not included in the present study). (B) Two-dimensional electrophoresis of BM plasma proteins (based on protocols we have previously described in detail (29)). Briefly, 13-cm strips (pH 3-10; GE Healthcare, USA) were rehydrated in a solution containing BM plasma proteins (300 µg) and subjected to isoelectric focusing. The second dimension was run as in (A). All 1-D and 2-D gels were stained with Coomassie blue and scanned at 300 dpi (Image Scanner, GE Healthcare, USA). Gel # 1 refers to BM sample from a patient with MDS with single lineage dysplasia (MDS-SLD); gels # 3, 6 and 7 refer to samples of BM plasma from MDS-RS patients; gels # 2, 4 and 5 contain BM plasma proteins from MDS-EB patients. [file Image_1.tif]
