# Supplementary material for: Global Proteomics Analysis of Bone Marrow: Establishing Talin-1 and Centrosomal Protein of 55 kDa as Potential Molecular Signatures for Myelodysplastic Syndromes
Source: Front Oncol. 2022 Jun 22;12:833068. doi: 10.3389/fonc.2022.833068 (PMC9257025; doi:10.3389/fonc.2022.833068)
Supplement: Supplementary file 3 [file Table_2.docx]

**Supplementary Table 2**. Proteins of the bone marrow plasma with different abundances in patients diagnosed with myelodysplastic syndrome with ring sideroblasts (MDS-RS) and patients with myelodysplastic syndrome with excess blasts (MDS-EB). Proteins were identified by label-free mass spectrometry and data, analyzed by Progenesis QI software. Data refer to patients listed in Table 1.

| **Accession number** | **Protein description** | **Overexpression in:** | **Max fold change** | **Confidence score*** | **Anova (p)** |
| --- | --- | --- | --- | --- | --- |
| Q53EZ4 | Centrosomal protein of 55 kDa OS=Homo sapiens GN=CEP55 PE=1 SV=3 | MDS-RS | Infinity | 24.72 | 0.003314 |
| 9ES2 | Inositol 1,4,5-trisphosphate receptor type 3 variant (Fragment) ,OS=Homo sapiens PE=2 SV=1 | MDS-EB | 56.65 | 30.24 | 0.008421 |
| P09871 | Complement C1s subcomponent | MDS-RS | 1.68 | 617.63 | 0.037679 |
| Q6ZP85 | cDNA FLJ26301 fis, clone DMC07540, OS=Homo sapiens PE=2 SV=1 | MDS-EB | 1.68 | 68.89 | 0.03393 |
| Q5NV69 | V1-13 protein (Fragment) | MDS-RS | 1.65 | 269.9 | 0.008245 |
| A0A087WW43 | Inter-alpha-trypsin inhibitor heavy chain H3 | MDS-RS | 1.61 | 851.32 | 0.001276 |
| B7Z392 | cDNA FLJ59305, highly similar to Paired amphipathic helix protein Sin3b ,OS=Homo sapiens PE=2 SV=1 | MDS-RS | 1.61 | 27.08 | 0.043766 |
| Q6PK75 | PRSS2 protein (Fragment) | MDS-RS | 1.59 | 165.52 | 0.009614 |
| E9PHK0 | Tetranectin | MDS-RS | 1.51 | 164.59 | 0.029409 |
| A0M8Q6 | Immunoglobulin lambda constant 7 | MDS-RS | 1.51 | 36.3 | 0.037644 |
| S6BGD6 | IgG L chain | MDS-EB | 1.50 | 2693.07 | 0.044683 |
| P43652 | Afamin | MDS-RS | 1.50 | 1266.44 | 0.0047 |
| B1N7B6 | Cryocrystalglobulin CC1 heavy chain variable region (Fragment) | MDS-RS | 1.43 | 111.61 | 0.024744 |
| P26038 | Moesin | MDS-EB | 2713.22 | 71.69 | 0.000174 |
| F5GZ39 | Polyubiquitin-C (Fragment) | MDS-EB | 2219.84 | 46.85 | 0.004344 |
| Q9Y490 | Talin-1 | MDS-EB | 294.77 | 268.42 | 0.022615 |
| B4DNW7 | Adenylyl cyclase-associated protein | MDS-EB | 193.84 | 117.91 | 0.019146 |
| A3R0T7 | Liver histone H1e | MDS-EB | 163.43 | 217.67 | 0.018475 |
| P06899 | Histone H2B type 1-J | MDS-EB | 138.86 | 47.59 | 0.013181 |
| P05164 | Myeloperoxidase | MDS-EB | 137.74 | 798.52 | 0.03517 |
| P16401 | Histone H1.5 | MDS-EB | 135.53 | 90.01 | 0.009784 |
| P12259 | Coagulation factor V | MDS-EB | 104.46 | 25.06 | 0.020701 |
| W8QEY1 | Lactoferrin | MDS-EB | 68.65 | 59.03 | 0.002933 |
| B4E022 | cDNA FLJ56274. highly similar to Transketolase (EC 2.2.1.1) | MDS-EB | 68.38 | 203.17 | 0.001416 |
| P24158 | Myeloblastin | MDS-EB | 62.89 | 117.02 | 0.048461 |
| P35579 | Myosin-9 | MDS-EB | 47.71 | 497.8 | 0.005338 |
| P08246 | Neutrophil elastase | MDS-EB | 34.97 | 253.31 | 0.008099 |
| Q6GMW3 | IGL@ protein | MDS-RS | 27.6 | 63.92 | 0.00095 |
| A2J1M8 | Rheumatoid factor RF-IP12 (Fragment) | MDS-RS | 25.93 | 40.95 | 4.88E-08 |
| B7ZLE1 | FRMPD4 protein | MDS-RS | 17.34 | 28.87 | 1.31E-06 |
| Q1HP67 | Lipoprotein. Lp(A) | MDS-RS | 17.08 | 60.99 | 0.041008 |
| S6AWF0 | IgG H chain | MDS-RS | 12.07 | 52.97 | 1.94E-06 |
| B4E1C2 | Kininogen 1. isoform CRA_b | MDS-EB | 11.23 | 32.27 | 0.009069 |
| Q68DX3 | FERM and PDZ domain-containing protein 2 | MDS-RS | 11.14 | 24.62 | 2.33E-07 |
| P01706 | Immunoglobulin lambda variable 2-11 | MDS-RS | 9.94 | 87.44 | 3.84E-05 |
| P01766 | Immunoglobulin heavy variable 3-13 | MDS-EB | 6.7 | 54.81 | 0.003265 |
| Q8NCL6 | cDNA FLJ90170 fis. clone MAMMA1000370. highly similar to Ig alpha-1 chain C region | MDS-RS | 6.11 | 56.17 | 1.96E-06 |
| Q0P5P4 | TMSB4X protein (Fragment) | MDS-RS | 6.01 | 108.96 | 0.037443 |
| A0A0C4DH72 | Immunoglobulin kappa variable 1-6 | MDS-EB | 5.89 | 31.18 | 0.001043 |
| A2IPI4 | HRV Fab 025-VL (Fragment) | MDS-RS | 4.94 | 160.31 | 0.000139 |
| S6B2A6 | IgG H chain | MDS-RS | 4.68 | 35.39 | 0.000352 |
| V9GYM3 | Apolipoprotein A-II | MDS-RS | 4.45 | 1017.33 | 5.20E-05 |
| S6BAP4 | IgG H chain | MDS-RS | 4.18 | 23.47 | 0.005491 |
| Q5FWF9 | IGL@ protein | MDS-RS | 3.67 | 92.15 | 0.001141 |
| S6BAN1 | IgG H chain | MDS-RS | 3.23 | 33.84 | 0.00937 |
| A0A0X9UWK7 | MS-D4 heavy chain variable region (Fragment) | MDS-RS | 2.97 | 55.64 | 0.003105 |
| A0A0X9T7T4 | MS-D4 heavy chain variable region (Fragment) | MDS-RS | 2.85 | 49.23 | 0.008127 |
| A0A0F7SZ86 | IGHV2-70 protein (Fragment) | MDS-RS | 2.84 | 88.51 | 0.01057 |
| Q9UL72 | Myosin-reactive immunoglobulin heavy chain variable region (Fragment) | MDS-RS | 2.79 | 26.88 | 0.012979 |
| A2J1N7 | Rheumatoid factor RF-ET10 (Fragment) | MDS-RS | 2.61 | 47.12 | 0.001045 |
| A0A1L2BU40 | Anti-staphylococcal enterotoxin E variable region lambda chain (Fragment) | MDS-RS | 2.59 | 70.74 | 0.002084 |
| A0A0C4DH42 | Immunoglobulin heavy variable 3-66 | MDS-EB | 2.57 | 27.42 | 0.021628 |
| S6AWG0 | IgG H chain | MDS-RS | 2.49 | 31.74 | 0.021142 |
| P20851 | C4b-binding protein beta chain | MDS-RS | 2.42 | 78.59 | 0.004515 |
| P51884 | Lumican | MDS-RS | 2.4 | 121.1 | 0.008007 |
| Q9NPP6 | Immunoglobulin heavy chain variant (Fragment) | MDS-RS | 2.38 | 75.38 | 0.006806 |
| A0A0B4J1X5 | Immunoglobulin heavy variable 3-74 | MDS-RS | 2.34 | 129.35 | 0.016739 |
| Q5W9F7 | KIAA0921 splice variant 1 (Fragment) | MDS-RS | 2.33 | 26.36 | 0.01116 |
| A0A0X9V9D6 | IBM-B2 light chain variable region (Fragment) | MDS-RS | 2.31 | 152.87 | 0.032806 |
| S6AWD9 | IgG H chain | MDS-RS | 2.31 | 146.67 | 0.011162 |
| A2NYV4 | Light chain Fab (Fragment) | MDS-RS | 2.07 | 67.92 | 0.016788 |
| Q9NZP8 | Complement C1r subcomponent-like protein | MDS-RS | 1.96 | 105.65 | 0.019833 |
| Q5VY30 | Retinol binding protein 4. plasma. isoform CRA_b | MDS-RS | 1.91 | 540.45 | 0.000593 |
| G3XAM2 | Complement factor I | MDS-RS | 1.83 | 489.9 | 0.002158 |
| P81605 | Dermcidin | MDS-RS | 1.78 | 117.57 | 0.011602 |
| V9GYE7 | Complement factor H-related protein 2 | MDS-RS | 1.76 | 151.94 | 0.008098 |
| E9KL23 | Epididymis secretory sperm binding protein Li 44a | MDS-EB | 1.74 | 7503.94 | 0.000979 |
| P02649 | Apolipoprotein E | MDS-RS | 1.72 | 796.9 | 0.017614 |
| Q9UL86 | Myosin-reactive immunoglobulin kappa chain variable region (Fragment) | MDS-RS | 1.69 | 82,46 | 0,027763 |
| A0A087X0Q4 | Immunoglobulin kappa variable 2-40 | MDS-RS | 1.42 | 112.32 | 0.016178 |
| Q15149 | Plectin | MDS-RS | 1.37 | 73.63 | 0.006839 |
| A2J422 | Anti-HER3 scFv (Fragment) | MDS-EB | 1.28 | 36.19 | 0.011048 |
| A0A068LKR4 | Ig heavy chain variable region (Fragment) | MDS-RS | 1.26 | 47.17 | 0.003049 |
