# Supplementary material for: Global Proteomics Analysis of Bone Marrow: Establishing Talin-1 and Centrosomal Protein of 55 kDa as Potential Molecular Signatures for Myelodysplastic Syndromes
Source: Front Oncol. 2022 Jun 22;12:833068. doi: 10.3389/fonc.2022.833068 (PMC9257025; doi:10.3389/fonc.2022.833068)
Supplement: Supplementary file 4 [file Table_3.docx]

**Supplementary table 3**. Quantitative data of *TLN1*, *MSN*, *CEP55* and *UBC* expression based on RT-PCR analysis of total RNA extracted from mononuclear cells of MDS patients depicted in Table 2.

| **mRNA *TLN1* expression (2^-∆Cq^)** | | | | | | | |
| --- | --- | --- | --- | --- | --- | --- | --- |
| **Chromosomal Abnormalities** | **N** | **Means** | **Standard Deviation** | **CI**  **Lower** | **CI**  **Upper** | **Levene**  **Test** | **p-value** |
| Normal Karyotype | 22 | .030816323 | .0238751394 | -.0180429148 | .0110031736 | 0.116 | 0.626 |
| Abnormal Karyotype | 15 | .034336193 | .0169139365 |  |  |  |  |
| **mRNA *MSN* expression (2^-∆Cq^)** | | | | | | | |
| **Chromosomal Abnormalities** | **N** | **Means** | **Standard Deviation** | **CI**  **Lower** | **CI**  **Upper** | **Levene**  **Test** | **p-value** |
| Normal Karyotype | 21 | .032498438 | .0246882095 | -.0253741839 | .0073130226 | 0.911 | 0.270 |
| Abnormal Karyotype | 16 | .041529019 | .0236779001 |  |  |  |  |
| **mRNA *CEP55* expression (2^-∆Cq^)** | | | | | | | |
| **Chromosomal Abnormalities** | **N** | **Means** | **Standard Deviation** | **CI**  **Lower** | **CI**  **Upper** | **Levene**  **Test** | **p-value** |
| Normal Karyotype | 24 | 0.004149 | 0.003211 | -0.004705 | -0.000060 | 0.264 | 0.045 |
| Abnormal Karyotype | 16 | 0.006532 | 0.004024 |  |  |  |  |
| **mRNA *UBC* expression (2^-∆Cq^)** | | | | | | | |
| **Chromosomal Abnormalities** | **N** | **Means** | **Standard Deviation** | **CI**  **Lower** | **CI**  **Upper** | **Levene**  **Test** | **p-value** |
| Normal Karyotype | 21 | .003518843 | .0035849090 | -.0010667465 | .0026811697 | 0.033 | 0.387 |
| Abnormal Karyotype | 16 | .002711631 | .0019365681 |  |  |  |  |
| **mRNA *TLN1* expression (2^-∆Cq^)** | | | | | | | |
| **WHO Classification** | **N** | **Means** | **Standard Deviation** | **CI**  **Lower** | **CI**  **Upper** | **Levene**  **Test** | **p-value** |
| MDS-RS | 13 | .470899862 | .2832045308 | -.0001784544 | .3421661459 | 0.114 | 0.049 |
| MDS-EB | 19 | .299906016 | .1921000511 |  |  |  |  |
| **mRNA *MSN* expression (2^-∆Cq^)** | | | | | | | |
| **WHO Classification** | **N** | **Means** | **Standard Deviation** | **CI**  **Lower** | **CI**  **Upper** | **Levene**  **Test** | **p-value** |
| MDS-RS | 13 | .028857562 | .0195627244 | -.0208844004 | .0082686076 | 0.998 | 0.384 |
| MDS-EB | 19 | .035165458 | .0200054812 |  |  |  |  |
| **mRNA *CEP55* expression (2^-∆Cq^)** | | | | | | | |
| **WHO Classification** | **N** | **Means** | **Standard Deviation** | **CI**  **Lower** | **CI**  **Upper** | **Levene**  **Test** | **p-value** |
| MDS-RS | 16 | .005680 | .003792 | -.002093 | .003106 | 0.952 | 0.695 |
| MDS-EB | 20 | .005173 | .003831 |  |  |  |  |
| **mRNA *UBC* expression (2^-∆Cq^)** | | | | | | | |
| **WHO Classification** | **N** | **Means** | **Standard Deviation** | **CI**  **Lower** | **CI**  **Upper** | **Levene**  **Test** | **p-value** |
| **MDS-RS** | 13 | .003535569 | .0030313032 | -.0029504801 | .0014180185 | 0.986 | 0.479 |
| **MDS-EB** | 18 | .004301800 | .0028636453 |  |  |  |  |
