# Supplementary material for: Global Proteomics Analysis of Bone Marrow: Establishing Talin-1 and Centrosomal Protein of 55 kDa as Potential Molecular Signatures for Myelodysplastic Syndromes
Source: Front Oncol. 2022 Jun 22;12:833068. doi: 10.3389/fonc.2022.833068 (PMC9257025; doi:10.3389/fonc.2022.833068)
Supplement: Supplementary file 5 [file Table_4.docx]

**Supplementary Table 4A**. Main functions of the microRNAs involved in the regulation of *TLN1* and *CEP55* based on the highest p-value obtained from miRNet database. (B) miRNAs functional enrichment associated with *TLN1* and *CEP55*, based on miRNet1 database.

***TLN1* 7**

| **Onco-MiRNAs** | **Cell Cycle** | **Bone Regeneration** | **Cell Death** | **Immune System** | **Adipocyte Differentiation** | **Angiogenesis** |
| --- | --- | --- | --- | --- | --- | --- |
| hsa-mir-24-3p | hsa-let-7b-5p | hsa-let-7b-5p | hsa-let-7b-5p | hsa-mir-25-3p | hsa-let-7b-5p | hsa-let-7b-5p |
| hsa-mir-92a-3p | hsa-mir-24-3p | hsa-mir-24-3p | hsa-mir-24-3p | hsa-mir-106a-5p | hsa-mir-92a-3p | hsa-mir-92a-3p |
| hsa-mir-222-3p | hsa-mir-92a-3p | hsa-mir-92a-3p | hsa-mir-25-3p | hsa-mir-17-3p | hsa-mir-222-3p | hsa-mir-222-3p |
| hsa-mir-373-5p | hsa-mir-222-3p | hsa-mir-222-3p | hsa-mir-92a-3p | hsa-mir-17-5p | hsa-mir-92a-2-5p | hsa-mir-92a-2-5p |
| hsa-mir-92a-2-5p | hsa-mir-373-5p | hsa-mir-92a-2-5p | hsa-mir-222-3p | hsa-mir-19a-3p | hsa-let-7a-5p | hsa-let-7f-5p |
| hsa-mir-106a-5p | hsa-let-7a-5p | hsa-mir-143-3p | hsa-mir-30c-2-3p | hsa-mir-20a-3p | hsa-let-7e-5p | hsa-mir-130a-3p |
| hsa-mir-107 | hsa-mir-103a-2-5p | hsa-mir-148b-3p | hsa-mir-30b-3p | hsa-mir-20a-5p | hsa-let-7f-5p | hsa-mir-149-5p |
| hsa-mir-146b-5p | hsa-mir-103a-3p | hsa-mir-17-3p | hsa-mir-30c-1-3p | hsa-mir-20b-5p | hsa-let-7g-5p | hsa-mir-15a-5p |
| hsa-mir-17-3p | hsa-mir-107 | hsa-mir-17-5p | hsa-let-7a-5p | hsa-mir-363-5p | hsa-let-7i-5p | hsa-mir-16-5p |
| hsa-mir-17-5p | hsa-mir-141-5p | hsa-mir-19a-3p | hsa-let-7c-5p | hsa-mir-93-3p | hsa-mir-103a-2-5p | hsa-mir-17-3p |
| hsa-mir-19a-3p | hsa-mir-143-3p | hsa-mir-20b-5p | hsa-let-7e-5p | hsa-mir-93-5p | hsa-mir-103a-3p | hsa-mir-17-5p |
| hsa-mir-19b-3p | hsa-mir-16-5p | hsa-mir-221-5p | hsa-let-7f-5p | hsa-mir-106b-5p | hsa-mir-130a-3p | hsa-mir-19a-3p |
| hsa-mir-20a-3p | hsa-mir-17-3p | hsa-mir-22-3p | hsa-let-7i-5p | hsa-mir-146a-5p | hsa-mir-143-3p | hsa-mir-19b-3p |
| hsa-mir-20a-5p | hsa-mir-17-5p | hsa-mir-22-5p | hsa-mir-143-3p | hsa-mir-155-5p | hsa-mir-17-3p | hsa-mir-20a-3p |
| hsa-mir-20b-5p | hsa-mir-182-5p | hsa-mir-23b-5p | hsa-mir-16-5p | hsa-mir-181b-5p | hsa-mir-17-5p | hsa-mir-20a-5p |
| hsa-mir-21-5p | hsa-mir-185-3p | hsa-mir-424-5p | hsa-mir-17-3p |  | hsa-mir-19a-3p | hsa-mir-21-5p |
| hsa-mir-221-5p | hsa-mir-185-5p | hsa-mir-93-3p | hsa-mir-17-5p |  | hsa-mir-20a-3p | hsa-mir-221-5p |
| hsa-mir-22-3p | hsa-mir-195-5p | hsa-mir-93-5p | hsa-mir-182-5p |  | hsa-mir-20a-5p | hsa-mir-22-3p |
| hsa-mir-22-5p | hsa-mir-19b-3p | hsa-mir-155-5p | hsa-mir-19b-3p |  | hsa-mir-221-5p | hsa-mir-22-5p |
| hsa-mir-93-3p | hsa-mir-20a-3p |  | hsa-mir-20a-3p |  | hsa-mir-27a-3p | hsa-mir-296-5p |
| hsa-mir-93-5p | hsa-mir-20a-5p |  | hsa-mir-20a-5p |  |  | hsa-mir-34a-5p |
| hsa-mir-106b-5p | hsa-mir-21-5p |  | hsa-mir-21-5p |  |  | hsa-mir-363-5p |
| hsa-mir-146a-5p | hsa-mir-221-5p |  | hsa-mir-221-5p |  |  | hsa-mir-370-3p |
| hsa-mir-155-5p | hsa-mir-29b-1-5p |  | hsa-mir-23b-5p |  |  | hsa-mir-497-5p |
| hsa-mir-27a-3p | hsa-mir-34a-5p |  | hsa-mir-29b-1-5p |  |  | hsa-mir-93-3p |
|  | hsa-mir-424-5p |  | hsa-mir-34a-5p |  |  | hsa-mir-93-5p |
|  | hsa-mir-503-5p |  | hsa-mir-98-5p |  |  | hsa-mir-146a-5p |
|  | hsa-mir-9-5p |  | hsa-mir-129-2-3p |  |  |  |
|  | hsa-mir-96-5p |  | hsa-mir-146a-5p |  |  |  |
|  | hsa-mir-98-5p |  | hsa-mir-181b-5p |  |  |  |

***CEP55* 6**

| **Apoptosis** | **Bone Regeneration** | **DNA Damage Response** | **Cardiotoxicity** | **Cell Cycle** | **Regulation of Akt Pathway** | **Onco-MiRNAs** |
| --- | --- | --- | --- | --- | --- | --- |
| hsa-mir-15a-5p | hsa-mir-19a-3p | hsa-mir-15a-5p | hsa-mir-199a-3p | hsa-mir-16-5p | hsa-mir-19a-3p | hsa-mir-19a-3p |
| hsa-mir-16-5p | hsa-mir-155-5p | hsa-mir-16-5p | hsa-mir-15b-5p | hsa-mir-19b-3p | hsa-mir-19b-3p | hsa-mir-19b-3p |
| hsa-mir-19b-3p | hsa-mir-130b-3p | hsa-mir-27a-3p | hsa-mir-130a-3p | hsa-mir-103a-3p | hsa-mir-155-5p | hsa-mir-27a-3p |
| hsa-mir-130a-3p | hsa-mir-148b-3p | hsa-mir-15b-5p | hsa-mir-424-5p | hsa-mir-15b-5p | hsa-mir-222-5p | hsa-mir-155-5p |
| hsa-mir-195-5p | hsa-mir-424-5p | hsa-mir-130a-3p | hsa-mir-199b-3p | hsa-mir-195-5p | hsa-mir-18a-3p | hsa-mir-222-5p |
| hsa-mir-155-5p | hsa-mir-222-5p | hsa-mir-16-1-3p | hsa-mir-182-5p | hsa-mir-424-5p | hsa-mir-196a-5p | hsa-mir-18a-3p |
| hsa-mir-424-5p | hsa-mir-196a-5p | hsa-mir-18a-3p | hsa-mir-199a-5p | hsa-mir-222-5p | hsa-mir-26a-5p | hsa-mir-196a-5p |
| hsa-mir-497-5p | hsa-mir-92a-3p | hsa-mir-196a-5p | hsa-mir-34a-5p | hsa-mir-16-1-3p | hsa-mir-92a-3p | hsa-mir-92a-3p |
| hsa-mir-222-5p | hsa-mir-23b-3p | hsa-mir-34a-5p | hsa-mir-214-3p | hsa-mir-182-5p | hsa-mir-205-5p | hsa-mir-107 |
| hsa-mir-144-3p | hsa-let-7b-5p | hsa-mir-34c-5p | hsa-mir-34c-5p | hsa-mir-18a-3p | hsa-mir-214-3p | hsa-mir-203a-3p |
| hsa-mir-16-1-3p | hsa-mir-1-3p |  |  | hsa-mir-196a-5p | hsa-mir-17-5p | hsa-mir-210-3p |
| hsa-mir-181a-5p | hsa-mir-210-3p |  |  | hsa-mir-34a-5p |  | hsa-mir-214-3p |
| hsa-mir-182-5p | hsa-mir-34c-5p |  |  | hsa-mir-92a-3p |  | hsa-mir-17-5p |
| hsa-mir-18a-3p | hsa-mir-17-5p |  |  | hsa-let-7b-5p |  | hsa-mir-93-5p |
| hsa-mir-26a-5p | hsa-mir-93-5p |  |  | hsa-mir-1-3p |  |  |
| hsa-mir-34a-5p |  |  |  | hsa-mir-107 |  |  |
| hsa-mir-92a-3p |  |  |  | hsa-mir-210-3p |  |  |
| hsa-mir-1-3p |  |  |  | hsa-mir-34c-5p |  |  |
| hsa-mir-126-3p |  |  |  | hsa-mir-449a |  |  |
| hsa-mir-203a-3p |  |  |  | hsa-mir-449b-5p |  |  |
| hsa-mir-210-3p |  |  |  | hsa-mir-17-5p |  |  |
| hsa-mir-449a |  |  |  |  |  |  |
| hsa-mir-449b-5p |  |  |  |  |  |  |

**Supplementary Table 4B**. miRNAs functional enrichment associated with *TLN1* and *CEP55*, based on miRNet1 database.

***TLN1***

| **Name** | **Hits** | **Pval** | **adj.Pval** |
| --- | --- | --- | --- |
| Onco-MiRNAs | 25 | 0.00000124 | 0.000124 |
| Cell Cycle | 30 | 0.0000539 | 0.0018 |
| Bone Regeneration | 19 | 0.000054 | 0.0018 |
| Cell Death | 30 | 0.000119 | 0.0027 |
| Immune System(Xiao's Cell2010) | 15 | 0.000135 | 0.0027 |
| Adipocyte Differentiation | 20 | 0.000398 | 0.006272727 |
| Angiogenesis | 27 | 0.0005 | 0.006272727 |
| Aging | 28 | 0.000597 | 0.006272727 |
| Hematopoiesis | 26 | 0.000623 | 0.006272727 |
| Latent Virus Replication | 12 | 0.000644 | 0.006272727 |
| Muscle Regeneration | 4 | 0.00069 | 0.006272727 |
| Cardiac Remodeling | 8 | 0.000975 | 0.008125 |
| T-helper 17 Cell Differentiation | 12 | 0.00191 | 0.01469231 |
| Cell Proliferation(Hwang Etal Bjc2007) | 5 | 0.00211 | 0.01486667 |
| Myogensis | 7 | 0.00223 | 0.01486667 |
| Anti-Cell Proliferation(Hwang Etal Bjc2007) | 7 | 0.00293 | 0.0183125 |
| Regulation of Akt Pathway | 14 | 0.00322 | 0.01894118 |
| Regulation of ATR Pathway | 3 | 0.00553 | 0.03068421 |
| T-Cell Differentiation | 10 | 0.00583 | 0.03068421 |
| Cell Division | 10 | 0.00792 | 0.0396 |
| DNA Synthesis | 3 | 0.0104 | 0.04952381 |

***CEP55***

| **Name** | **Hits** | **Pval** | **adj.Pval** |
| --- | --- | --- | --- |
| Apoptosis | 23 | 0.0000431 | 0.00249 |
| Bone Regeneration | 15 | 0.0000498 | 0.00249 |
| DNA Damage Response | 10 | 0.000228 | 0.0076 |
| Cardiotoxicity | 10 | 0.000327 | 0.008175 |
| Cell Cycle | 21 | 0.000416 | 0.00832 |
| Regulation of Akt Pathway | 11 | 0.00257 | 0.03525 |
| Onco-MiRNAs | 14 | 0.00279 | 0.03525 |
| Toxicity | 13 | 0.00282 | 0.03525 |
| T-helper 17 Cell Differentiation | 9 | 0.0035 | 0.03888889 |
| Peritoneal Cavity Homeostasis(26495316) | 10 | 0.00406 | 0.03953846 |
| Endocytosis | 5 | 0.00439 | 0.03953846 |
| Angiogenesis | 18 | 0.00509 | 0.03953846 |
| Cell Death | 19 | 0.00514 | 0.03953846 |
